# Training and support for caring for a child’s gastrostomy: a survey with family carers

**DOI:** 10.1136/bmjpo-2021-001068

**Published:** 2021-07-27

**Authors:** Bethan Page, Siobhan Butler, Colette Smith, Alex CH Lee, Charles A Vincent

**Affiliations:** 1 Experimental Psychology, University of Oxford, Oxford, Oxfordshire, UK; 2 Helen and Douglas House, Oxford, Oxfordshire, UK; 3 Oxford Health NHS Foundation Trust, Oxford, UK; 4 Oxford University Hospitals NHS Foundation Trust, Oxford, UK

**Keywords:** gastroenterology, health services research, nursing

## Abstract

**Objective:**

The aim of this study was to explore family carers’ experiences of training and ongoing support for caring for their child’s gastrostomy, and to get their views on how this could be improved.

**Methods:**

A mixed-methods online survey with 146 family carers (eg, parents, grandparents) who care for a child with a gastrostomy. Family carers rated their own experience of training and support and made recommendations for how training and support could be improved for future families.

**Results:**

The nature and extent of the training family carers reported receiving varied considerably. Many felt that the demonstrations they received in hospital were too brief. Two in five family carers rated their confidence caring for their child’s gastrostomy as very low in the first few weeks after surgery. Parents valued ongoing learning and support from other parents and support from community nurses. Videos and simulation practice were rated as useful formats of training, in addition to face-to-face supervised practice with a clinician. Parents liked how real life the example video shown was, and rated nearly all suggested video topics as ‘very helpful’, especially troubleshooting topics.

**Conclusions:**

Our study found substantial variability in family carers’ descriptions of the training and support they received to care for their child’s gastrostomy. Training often did not meet family carers’ needs. We need to invest in better training and support for families and learn from their recommendations. Improvements to training and support for families (eg, through instructional videos) have the potential to improve family carers’ confidence and competence, and reduce the risk of problems and complications which cause harm to children and increase demand on National Health Service (NHS) resources.

**What is known about the subject?**

- It is now commonplace for families to carry out complex medical procedures for children at home.
- Some studies have reported safety concerns around the practices of parents caring for children with gastrostomies.

**What this study adds?**

- The training family carers report receiving to care for their child’s gastrostomy varies considerably and often does not fully meet their needs.
- Many family carers report feeling anxious and under-confident in the first few weeks at home after their child’s surgery.
- Family carers value supervised practice with healthcare professionals, videos featuring parents and clinicians and more hands on practice, including using models/dolls.
- Training should begin prior to the hospital admission for surgery and be regarded as an essential component of the care package.

## Introduction

Many medical procedures, which were once carried out only by healthcare professionals on hospital wards, are now performed by families at home.^1–3^ There are increasing numbers of children with complex medical needs.^4 5^ Their parents perform a range of medical procedures for them at home, including feeding tube care, home oxygen, tracheostomy care and bowel washouts.^1 4 6^ There are numerous benefits for families and the healthcare system when children can be cared for at home, rather than in healthcare settings,^7 8^ but there are also substantial risks.^2^ An analysis of incident reports on enteral tube feeding in the UK identified various safety concerns relating to training and support for parents.^9^ Without consistent high-quality training and support for families, children are at risk of harm, and families feel under-confident, anxious and alone.^6 10^

### Caring for a child with a gastrostomy

A gastrostomy is a surgically placed device where feeds are delivered directly into the stomach. Gastrostomies are common in children with severe chronic illnesses and neurodisability who have difficulty swallowing or cannot get adequate nutrition through eating and drinking.^11 12^ Family carers (eg, parents) learn to administer feeds, water and medications via the tube, to clean and care for the stoma site, and to manage complications such as leakage and blocked tubes. Family carers may also learn to change a gastrostomy button. Family carers normally receive some bedside teaching in hospital (during a hospital stay of around 2 days), with ongoing support from community nurses, but this may not be sufficient to ensure safe care at home. There are some safety concerns reported in the literature around the practices of parents caring for children with gastrostomies.^9 13 14^ In a recent study on same-day discharge for gastrostomy surgery, the most common reason for a delayed discharge was parents not feeling sufficiently confident caring for the gastrostomy.^15^ Improving training and support for families could reduce callouts to overstretched community teams, as well as preventing avoidable Emergency Department visits and admissions, for example, through appropriate timely management of a dislodged gastrostomy button.

Many parents caring for children with complex medical needs develop substantial expertise over time.^6 9 10^ However training for families can be informal and highly variable.^6 10 11^ This contrasts with training for healthcare professionals who receive years of university training, practice-based training and ongoing continuing professional development. Healthcare professionals typically also have on-site backup and support, whereas families are often home alone while performing medical tasks.^16^ Good quality training for family carers is key for optimising outcomes for children.^17^ The aim of this study is to explore family carers’ experiences of training and ongoing support for caring for their child’s gastrostomy, and to find out their views on how training and support could be improved. This paper is a part of a longer-term project to develop a package of training and support for families.^18^

## Methods

### Survey aims

The first aim was to understand family carers’ experiences of training and support for caring for their child’s gastrostomy. The second aim was to understand family carers’ recommendations for improving training and support.

### Survey development

The survey was developed in stages. In the initial exploratory stage, we reviewed the literature on gastrostomy training and consulted parent representatives, nurses and paediatricians from the community and hospital. The content of the survey was informed by the findings from a preliminary qualitative survey with 50 families who performed a range of medical procedures, which highlighted family carers’ feelings of being scared and unprepared, the variability in experiences of training and the emotional demands on families.^19^ The survey instrument was drafted, piloted and revised in consultation with parent representatives, children’s nurses and paediatricians from the community, a paediatric gastrointestinal surgeon and a specialist surgical nurse. The team was asked to comment on the suitability of the questions, readability and length. Recruitment strategies were developed based on advice from our parent representatives, clinicians and charities.

### Survey design and content

The survey was a mixed-methods survey with the qualitative data intended to complement, illustrate and expand the quantitative data.^20^ The survey tool used was Qualtrics. The survey is available in online supplemental file 1.

10.1136/bmjpo-2021-001068.supp1 Supplementary data

### Sampling and recruitment

Participants were recruited through UK charities and local charities and through our parent representatives who posted on closed Facebook groups which serve as support groups for families (‘Tube Feeding your child in the UK’ which has 4105 members, and the ‘Blended Diet UK’ group which has 4200 members). The sample is best described as a convenience sample, however we purposely advertised through charities that support children with a range of different diagnoses and levels of complexity (eg, Well Child, TOFS, Together for Short Lives) and sought to recruit family carers with different levels of experience (assessed as number of years since the child’s gastrostomy surgery). It was clear from the survey question ‘where did you hear about the survey’ that some people also chose to share the survey with friends/family. The advertisement information informed participants that we were looking for family carers (eg, parents) who cared for children with gastrostomies to complete a survey on their training and support needs. The exact wording varied slightly between the different charities and posts on Facebook groups. The first page of the survey gave some brief information about the survey (see online supplemental file 1).

The inclusion criteria were any parent or family carer who provides gastrostomy care at home to a child or young person aged under 25 years. By family carer, we included any unpaid carer (parent, relative, friend) who actively participates in caring for a child with a gastrostomy. To take part family carers needed to be at least 18 years old. Participants received a £10 voucher for taking part. All participants gave informed consent before taking part and consented for their data to be used in publications (The Medical Sciences Inter-Divisional Research Ethics Committee, Oxford University, R56623/RE004). The data were collected between July and September 2020. We aimed to recruit at least 100 participants to capture a broad range of experiences, including family carers new to gastrostomy care and some more experienced.

### Analysis

Descriptive statistics were computed for all close-ended questions, using SPSS Statistics V.25. Participants who did not fully complete the survey (defined as viewing all pages of the survey and completing all the quantitative questions at a minimum) were excluded. The open-ended questions were coded in NVivo V.12 using inductive content analysis, to group responses based on surface level of meaning.^21^ Answers were coded line by line, and grouped into categories emerging from the data. These were summarised and illustrated with quotes from participants.

### Patient and public involvement

Two parent representatives were involved in the design, conduct and dissemination of the research. The two parents attended the research meetings from conception of the project, alongside a team of multidisciplinary healthcare professionals. The recruitment strategy was devised through consultation with our parent representatives. The parents completed the draft survey, which was then revised based on their feedback and feedback from clinicians. The recommendations from the survey were developed through meetings with parents and the healthcare professionals supporting the research.

## Results

### Participants

One hundred and forty-six participants fully completed the survey. A total of 195 participants consented to take part and 250 responders viewed the first page. The majority of the 146 participants were mothers (91%). There was a range of ages of the children and time since initial gastrostomy surgery. The most common types of devices were gastrostomy buttons and percutaneous endoscopic gastrostomy tubes. Table 1 gives more details about the participants.

**Table 1:** The participants who completed the full survey

	N (%)
Relation to child	
Mother	133 (91%)
Father	8 (6%)
Other family member	5 (3%)
Age of participants’ children (years)	
0–4	50 (34%)
5–9	38 (26%)
10–14	39 (27%)
15–19	18 (12%)
20–25	1 (1%)
Time since initial gastrostomy surgery (years)	
<1 year	15 (10%)
1–2	41 (28%)
3–4	27 (18%)
5+years	63 (43%)
Type of gastrostomy device that the child has or previously had *	
PEG tube	73 (50%)
Gastrostomy button (MINI or Mic-Key)	115 (79%)
Another device (eg, GJ tube)	23 (16%)

*Some children had more than one gastrostomy device, for example, some children had a PEG tube initially which was later changed to a gastrostomy button.

GJ, gastrojejunostomy; PEG, percutaneous endoscopic gastrostomy.

### Participants’ descriptions of their training

Participants’ experiences of training were variable (see box 1). Most described receiving some training in hospital but the nature and extent of this training varied considerably. Descriptions of training as ‘*brief’* or ‘*basic’* were common. Some participants felt unprepared and anxious: ‘*It was scary because we were worried about it getting caught/pulled and hurting or causing damage to our little boy. We didn’t get much practice before being left to do it on our own so you are triple checking yourself and worrying did I do it right’.* Some participants described receiving further training at home by community nurses or representatives from a feeding company, as well as learning through other parents, often through Facebook groups: ‘*Also had amazing advice from other mums whose children have tubes who I found via Facebook groups - game changing stuff’.*

Box 1 **Example quotes illustrating the variability in experiences of training**

**Training sometimes brief and basic:**

- ‘A brief 5 min of basics, a leaflet and home. A few weeks later a Nutricia nurse came to the house to show us the pump’.
- ‘We were admitted for the surgery and spent two further days in the ward after, received very basic training on the use of the peg and left feeling absolutely terrified about using it!’
- ‘Need much more training than 10 min in consultant’s office!’

**Primarily self-taught with support from other parents:**

- ‘The official training—nothing good. Absolutely disastrous, vague, unsupportive. The unofficial training and the info I sought out for myself—clear, helpful videos from other parents, useful approach of gastrostomy nurse. Properly child-centric, helpful stuff’.

**More community support needed:**

- ‘The community nursing team rely too much on the surgical nurse to do the training and then they just catch up with a chat following any training given at the bedside. This training is not detailed/long enough for those dealing with such a complex medical needs child’.

**Thorough training and support received:**

- ‘Our daughter was in hospital long terms due to a range of factors. We were able to observe nurses undertaking feeds, using pump, giving medication. We were also given an information pack and work book to go through, and we were observed by nurses until confident and competent to do ourselves. We were given support in community…with regards to changing button, taking care of button, and annual refresher also. Received updates with regards to how much water to inflate balloon with, frequency of Ph testing etc’.

The majority of participants reported receiving some training from their hospital team (n=115, 79%). A slightly smaller proportion received training from a Community Children’s Nurse (CCN) (n=105, 72%). Thirty-five (24%) participants mentioned training from another provider, most commonly a feeding company (eg, Nutricia). Eighty-two (56%) participants reported receiving training from both the hospital and CCN team, and eight (5%) reported not receiving training from either the hospital team or a CCN team.

Table 2 shows the types of training participants received. The most common types of training were verbal information, demonstrations and supervised practice from a healthcare professional. Few participants received any simulation practice (hands-on practice with a doll/equipment), or watched instructional videos. A few of the participants who did report receiving simulation practice commented on the usefulness of this: ‘*Really useful being able to have a little play with a tube and practice using the clip etc. before having to do it for real*’.

**Table 2:** Types of teaching/training received by parents

Types of teaching/training received	N (%)
Given information verbally by a healthcare professional	129 (88%)
Demonstrations by a healthcare professional	125 (86%)
Practised on my child supervised by a healthcare professional	113 (77%)
Given a written booklet	85 (58%)
Simulation practice (practised with a doll or some equipment)	19 (13%)
Directed to a website for information	13 (9%)
Demonstrations by another family member	7 (5%)
Other	5 (3%)
Videos	3 (2%)

### Confidence over time

Participants were asked how confident they felt caring for their child’s gastrostomy in the first few weeks after surgery: 24 (16%) were not at all confident, 32 (22%) were slightly confident, 30 (21%) were moderately confident, 42 (29%) were mostly confident and 18 (12%) of participants said they were fully confident. At the time of the survey a majority (n=117, 80%) said they felt fully confident caring for their child’s gastrostomy. The most common concerns that participants reported were around hurting their child, caring for site after surgery, knowing ‘*what was normal’* in relation to the stoma site healing, worries about the tube coming out and problems such as blocked tubes or overgranulation: ‘*The first time I experienced this [granulomas] I thought my sons intestines were coming out! Nobody had ever mentioned it to me nor had I ever seen anything like it!’*

It is not possible to say from the data whether the training that participants received had improved over time. However, we did not find any evidence suggesting that participants’ retrospective confidence ratings from the first week at home had changed over time, which may suggest that training has not changed or improved over time: 40% of participants with less than a year’s experience rated themselves as ‘not at all confident’ or ‘slightly confident’ in the first week at home, compared with 38% of participants with more than 5 years experience. There was no statistically significant association between time since gastrostomy surgery and participants’ ratings of confidence in the first week at home: χ^2^(12, n=147)=12.06, p=0.44. Again, this may suggest that training has not changed or improved over time.

However, there was evidence that participants’ ratings of their current confidence (as rated at the time of the survey) did improve with more years of experience: 46% of participants with less than a years’ experience rated themselves as fully confident caring for their child’s gastrostomy compared with 89% of participants with more than 5 years experience. A χ^2^ test revealed a significant association between current confidence ratings and number of years since gastrostomy surgery: χ^2^(9, n=147)=17.54, p=0.04.

### Ongoing support and training

Participants were asked which sources were most helpful for ongoing support (see table 3). Contacting your community nurse was rated as ‘very helpful’ by a majority, with less than a quarter rating hospital teams as a very helpful source of support. Notably conversations with other parents was rated as ‘very helpful’ by 56%, with a further 32% rating support from parents as ‘quite helpful’. Participants in the survey offered advice for other parents, including tips for managing their child’s distress when changing a gastrostomy button.

**Table 3:** Sources of ongoing support: how helpful are they?

	Very helpful	Quite helpful	Not very helpful	Not applicable
Contacting your community nurse	90 (62%)	28 (19%)	13 (9%)	15 (10%)
Contacting your hospital team	34 (23%)	37 (25%)	38 (26%)	37 (25%)
Facebook groups	54 (37%)	67 (46%)	11 (8%)	14 (10%)
Conversations with other parents of children with similar needs	82 (56%)	47 (32%)	3 (2%)	14 (10%)
Written information booklets provided by a healthcare professional	28 (19%)	68 (47%)	32 (22%)	18 (12%)
NHS websites	15 (10%)	55 (38%)	52 (36%)	24 (16%)
Charities	25 (17%)	53 (36%)	19 (13%)	49 (34%)
Videos	29 (20%)	50 (34%)	17 (12%)	50 (34%)

Participants were asked, at the time of completing the survey, whether further training might be helpful to them: 14 (10%) said definitely yes to further training or support, 29 (20%) probably yes, 81 (55%) probably not and 21 (14%) definitely not. The most common request was help with managing problems. A number of participants wanted refresher training, or updates on the latest guidance: ‘*making sure bad habits have not crept in and that we are up-to-date with any changes in how things are done*’.

### Participants’ recommendations for improving training

Participants were asked which formats of training might be useful to other parents facing the challenge of caring for a child with a gastrostomy (see table 4). Around three-quarters rated demonstrations and practice on your child supervised by a healthcare professional as extremely useful. Around two-thirds of participants felt that videos and simulation practice would be extremely or very useful. Participants felt that these additional forms of training would be a useful addition to face-to-face training with a professional, but not a substitute: ‘*I think that the videos are good resources that parents can go back to however I think that face to face training is really important initially*’.

**Table 4:** Types of training that might be helpful to other parents

	Extremely useful	Very useful	Moderately useful	Slightly useful	Not at all useful
Demonstrations by a healthcare professional	105 (72%)	33 (23%)	6 (4%)	2 (1%)	0 (0%)
Practising on your child supervised by a healthcare professional	110 (75%)	29 (20%)	5 (3%)	1 (1%)	1 (1%)
Written booklets	32 (22%)	39 (27%)	51 (35%)	19 (13%)	5 (3%)
Videos	40 (27%)	58 (40%)	29 (20%)	14 (10%)	5 (3%)
Simulation training (practiing with a doll or some equipment)	47 (32%)	45 (31%)	36 (25%)	10 (7%)	7 (5%)
Online training	18 (12%)	33 (23%)	47 (32%)	30 (21%)	17 (12%)
Group training with other parents	20 (14%)	34 (23%)	39 (27%)	31 (21%)	21 (14%)
Training by experienced parents	15 (10%)	16 (11%)	32 (22%)	51 (35%)	32 (22%)

Participants had many suggestions for improving training. A common suggestion was more training and information about common problems including what to do if the button comes out, and requests for specific types of training, such as more hands-on training. Some participants commented on the timing of training as they struggled to take information on board while in hospital: ‘*I think we should have had more [training] before his surgery and not while he was in theatre as we were so anxious I’m not sure how much we took in or how valuable doing it then was*’. A few participants said they would like to have been put in touch with other families: ‘*Opportunity to do group talks or training would be good. Nice way to meet other families and build a support network*’.

### Recommendations for developing training videos

Participants watched a sample video showing a parent administering a bolus of water to their child. Participants liked how ‘real life’ the video was: the child was wriggling during the procedure, it was done at home, it was relaxed and the mother was talking to the child throughout. One participant commented that: ‘*I've hated the teaching videos previously as they seem rather clinical but this was fantastic*’. When asked where training videos should be filmed, 54 (37%) said at home, 4 (3%) said in hospital and 88 (60%) said a mixture of both locations. A total of 116 (80%) participants wanted both healthcare professionals and parents to feature, 12 (18%) wanted just healthcare professionals and 8 (12%) wanted just parents. Participants rated different topics for videos: the vast majority were rated as ‘very helpful’ (see online supplemental file 2). More specific recommendations from participants for developing training videos are available in online supplemental file 2.

10.1136/bmjpo-2021-001068.supp2 Supplementary data

## Discussion

Family carers’ descriptions of the training they received to care for their child’s gastrostomy varied considerably. Demonstrations in hospital were frequently described as too brief and insufficient to prepare families. Many family carers reported feeling anxious in the first few weeks of caring for their child at home and concerns about doing something wrong or hurting their child. Most family carers however reported feeling confident to care for their child’s gastrostomy at the time of the survey. Family carers particularly valued ongoing support from other parents and from community nurses. Videos and simulation practice were rated as useful preparation, in addition to face-to-face supervised practice with a healthcare professional. Participants wanted videos to feature parents and healthcare professionals and for at least some of the videos to be filmed at home.

### Strengths and limitations

This study has a number of strengths. Parents with different levels of experience responded to the survey, from families who were very new to gastrostomy care to families with more than 5 years' experience. Families were recruited through charities and Facebook support groups whose members come from across the UK: this suggests that the issues described by families in the survey are not unique to one region or service. Families were very engaged and many offered to help support the development of training videos and other resources.

One key weakness of the study is that we did not collect demographic data on the families so cannot tell the socioeconomic, health literacy or ethnicity of families, or information about the children’s diagnoses or their level of medical complexity. It is therefore impossible to know how selection bias played out in our study. We may have recruited families who are more engaged in their child’s care or families who felt unprepared and sought help through Facebook groups and charities. It is possible there are some issues of recall in family carers who received their training a long time ago (43% of parents had more than 5 years’ experience of caring for their child’s gastrostomy).

### Implications for the design of services

Good quality training for family carers is recognised in the literature as key for optimising outcomes for children and preventing harm.^14 17^ Shorter hospital stays are increasingly advocated for gastrostomy surgery, including same-day discharges,^15^ meaning that there is little time to train families during admission. Many family carers in our survey reported not feeling confident caring for their child in the first few weeks at home after surgery. Other studies have similarly reported concerns that some families do not feel confident to care for their child’s gastrostomy on discharge from hospital,^15^ as well as gaps in knowledge and inadequate skills in some family carers.^13 14^ Our study documents some of family carers’ concerns and also importantly their recommendations for improving training and support. Family carers may benefit from more preparation for caring for their child’s gastrostomy (from either hospital or community nurses) before surgery takes place: this has the potential to improve confidence ratings in the first few weeks at home after surgery.

Box 2 gives an overview of practical suggestions for improving training based on discussions of the survey data with our multidisciplinary group of healthcare professionals and parents. Family carers may benefit from viewing instructional videos and written materials before the surgery with the resources available to revisit as needed. To enable more opportunity for hands-on practice, family carers could practice with dolls and equipment: there is substantial evidence on the benefits of repeated hands-on practice in the medical education literature.^22 23^ There is potential to make more use of online training and support, including group video calls for families. Training resources should be codesigned with families and address their emotional needs (such as recognising parental anxiety and fears and discussing the potential impact of a gastrostomy on daily life) as well as the technical aspects of care.

Box 2 **Practical suggestions for improving training based on discussions on the survey findings with a multidisciplinary group of healthcare professionals and parents**

**Before surgery**

- Family carers may benefit from videos teaching them about routine care of a gastrostomy, how to manage common problems and advice and tips from more experienced families. Families recommend videos should feature both parents and healthcare professionals and some should be filmed at home. It is also important for the videos to feature families from different cultural backgrounds and to be accessible to families who do not speak fluent English.
- Family carers may benefit from a home visit (eg, from a community nurse) for face-to-face teaching and the opportunity to ask questions.
- Family carers may value repeatedly practicing with dolls/equipment to become familiar with the basics before their child’s surgery, for example, connecting the extension tube.
- Family carers could be invited to a group call with other families awaiting surgery to ask questions to the surgical team and meet other parents.
- Family carers may benefit from recommendations for Facebook groups to join and other peer support options.

**During hospital admission**

- Family carers highly value supervised practice doing procedures on their child after surgery with the support of a designated nurse. It is important that adequate time is given to this important part of training.

**After hospital admission**

- Family carers will likely benefit from further support from community nurses who can provide further teaching and support through home visits and video calls.
- Family carers will likely benefit from revisiting videos and written materials as needed, including videos on managing common problems such as overgranulation or blocked tubes.
- Family carers need to be supported by community nurses to learn to change a button, and may benefit from repeated practice with models in addition to supervised changes on their child.

### Future research and development

Our multidisciplinary group is currently creating a library of videos coproduced with families, paediatricians and nurses from the hospital and community and piloting hands-on practice with equipment and 3D-printed models. The videos and further information about the programme are available online.^18^ Evaluation of the videos is ongoing. Future research is needed to better understand healthcare professionals’ views on training families and ideas for improvement, to compare against the findings from the families in this survey. It is also important to explore the experiences of training in family carers with low health literacy or with limited English language.

## Conclusions

Our study found substantial variability in family carers’ descriptions of the training and support they received to care for their child’s gastrostomy. Many felt that the training they received did not prepare them sufficiently. Family carers valued face-to-face training with a healthcare professional, videos which show ‘real-life’ featuring families and clinicians and hands-on practice including with dolls/equipment. We need to invest in better training and support for families and learn from their recommendations. Improvements to training and support for families have the potential to improve family carers’ confidence and competence, and reduce the risk of problems and complications which cause harm to children and increase demand on NHS resources.

## Supplementary Material

Author's manuscript

## Data Availability

Data are available upon reasonable request. Data may be obtained upon reasonably request by contacting the corresponding author. The survey tool used is available in the supplemental information.
