# Supplementary material for: Training and support for caring for a child’s gastrostomy: a survey with family carers
Source: BMJ Paediatr Open. 2021 Jul 27;5(1):e001068. doi: 10.1136/bmjpo-2021-001068 (PMC8317118; doi:10.1136/bmjpo-2021-001068)
Supplement: Supplementary data [file bmjpo-2021-001068supp001.pdf]

Supplementary File 1: Survey Tool

Overview of survey

Gastrostomy care: an online survey for parents on training and support needs

- This survey is for family carers (e.g. mums, dads, grandparents) who care for a child or young person who has a gastrostomy (e.g. PEG or button).
- This survey is part of a project to develop resources to better prepare and support families who are new to gastrostomy care. The project is a collaboration between researchers, parents and healthcare professionals.
- The findings will be shared widely with parents, charities, healthcare providers and researchers.

What do I have to do to take part?

- This study is an online survey and will take 15-20 minutes to complete.
- You will be asked about your experiences of learning to care for your child's gastrostomy, your experiences of ongoing support and what videos and resources might be helpful.
- To take part, the child you care for needs to be under 18 years and you need to be at least 18 years old.
- Please read the participant information sheet on the next page if you are interested in taking part.

Thank you for your time.

Page Break

Participant information

We [the University of Oxford in collaboration with clinicians and parent representatives] are investigating the experiences and training needs of family carers in the UK who provide specialist healthcare to children at home, in order to develop resources for training and supporting other families. We are currently creating videos and resources for parents new to gastrostomy care and are seeking your feedback on how we should do this.

We appreciate your interest in participating in this online survey. You have been invited to participate as you are a parent or a family carer who provides gastrostomy care to a child or young person under 18 at home. By family carer, we include any unpaid carer (parent, relative, friend, foster carer) who actively participates in caring for a child or young person with a gastrostomy. Tasks you might do include giving feeds, medications, caring for the stoma site and/or changing a gastrostomy button. To take part you, the family carer, need to be at least 18 years old.

Please read through this information before agreeing to participate by ticking the 'yes' box below.

You will be asked questions on:

i) you and your child (e.g. child's age, your relationship to the child)

ii) your experiences of learning and being trained to care for your child's gastrostomy

iii) your experiences of ongoing support with caring for your child's gastrostomy

iv) what training and support would help other parents

v) what videos would be helpful

The survey should take 15-20 minutes to complete. No background knowledge is required.

How will your data be used?

The survey will be used to inform the development of videos and other resources to support and prepare families new to gastrostomy care. Some of the videos will also be helpful to more experienced parents and for training staff.

The study results will be written up in a DPhil (PhD) thesis and potentially also in an academic publication. Key findings will be shared widely with participants, family carers, charities, health care professionals and researchers.

Your answers will be completely anonymous, and we will use all reasonable endeavours to keep them confidential. You will be given the option at the end of the survey to provide us with your email address if you would like to receive a summary of the research findings or if you would be interested in helping us rate and evaluate the videos we are creating. Email addresses and telephone numbers will be stored on a password protected file on a secure university server. This is part of a longer term project to develop new methods for training and supporting family carers.

Please note that your participation is voluntary. You may withdraw at any point during the survey for any reason, before submitting your answers, by closing the browser. If you need to leave the survey before you have time to fully complete it, you can return to finish your partially completed survey using the same device (tablet, computer, mobile) within one week of starting it. After one week, your data will be saved and you will be unable to continue the survey.

Your data will be stored in a password-protected file on secure university servers and may be used in academic publications. Your IP address will not be stored. All questions are optional.

Who will have access to your data?

The University of Oxford is the data controller for the purposes of the Data Protection Act 1998. Your information may be shared with members of the research team. This questionnaire is for a DPhil [PhD] project. The principal researcher is Bethan Page, who is attached to the Department of Experimental Psychology at the University of Oxford. This project is being completed under the supervision of Professor Charles Vincent, Professor Nick Yeung and Dr Emily Harrop (clinical lead for the project).

This project has been reviewed by, and received ethics clearance through, the University of Oxford Central University Research Ethics Committee [reference number R56623/RE001].

Participant benefits and risks

If you complete the full survey, we will email you a £10 Marks and Spencer (M&S) voucher as a thank you for taking part. You will be asked to leave your email address at the end of the survey so we can send you the voucher. Your email address will be deleted from our records after we have sent you the voucher (unless you have consented to be contacted in the future with a summary of the findings, or to help with future studies).

There will be no other direct benefits to you in taking part in this study. However, you may find it useful to share your experiences and views on improving training and support. Your involvement will improve our understanding of current training provision and support needs of family carers. Key findings will be used to develop resources to better support and prepare family carers, and will be shared widely with families, healthcare providers, charities and researchers.

There are no direct risks involved in taking part in this study. However it is possible that you may find some of the topics distressing or difficult. You don’t have to answer all the questions and you can exit the survey at any point or withhold any information that you do not wish to share. A list of website and charities offering further support will be provided at the end of the survey.

What if there is a problem?

If you have a concern about any aspect of this project, please contact the researcher Bethan Page (email: bethan.page@psy.ox.ac.uk) or their supervisor Professor Charles Vincent (charles.vincent@psy.ox.ac.uk) who will do their best to answer your query. If your problem is a clinical query, we will contact the clinical lead for the project, Dr Emily Harrop (Consultant in Paediatric Care) for guidance.

The researcher should acknowledge your concern within 10 working days and give you an indication of how they intend to deal with it. If you remain unhappy or wish to make a formal

complaint, please contact the relevant Chair of the Research Ethics Committee at the University of Oxford:

Chair, Medical Sciences Inter-Divisional Research Ethics Committee;

Email: ethics@medsci.ox.ac.uk;

Address: Research Services, University of Oxford, Wellington Square, Oxford OX1 2JD

The Chair will seek to resolve the matter in a reasonably expeditious manner.

If you have any questions about the survey please email bethan.page@psy.ox.ac.uk.

Please note that you may only participate in this survey if you are 18 years of age or over.

○ I am 18 years or over

If you have read the information above and agree to participate with the understanding that the data (including any personal data) you submit will be processed accordingly, please check the relevant box below to get started.

○ Yes I agree to take part

Page B, *et al. BMJ Paediatrics Open* 2022; 5:e001068. doi: 10.1136/bmjpo-2021-001068

About you and your child

Section 1 of 5:
In this first section we would like to ask some questions about you and the child or young person you provide care for.

Q1.1 What is your relationship to the child that you care for?

○ Mother (1)

○ Father (2)

○ Other [please specify] (4) ____

Q1.2 How old is your child?

____

Q1.3 How long ago did your child first have gastrostomy surgery?

○ Less than a year (1)

○ 1 - 2 years (2)

○ 3 - 4 years (3)

○ 5 + years (4)

Q1.4 Which types of gastrostomy tube has your child had? [select all that apply]

- ☐ Gastrostomy button (e.g. MIC-KEY button, MINI button) (1)
- ☐ Percutaneous Endoscopic Gastrostomy (PEG) (e.g. Freka, Corflo) (2)
- ☐ Other [please specify] (3) ____

Experiences of learning and being trained

Section 2 of 5:
In this section we would like to understand what training and information you received to care for your child's gastrostomy and what you found helpful.

Parents have to learn to give feeds and medications, clean and care for the stoma site and learn what to do if there is a problem, such as if the PEG tube blocks or the gastrostomy button comes out. There are also other tasks you may have learnt to do such as changing a gastrostomy button.

Q2.1 Tell us about what training and information you received when your child first had gastrostomy surgery.

____

____

____

____

____

Q2.2 Tell us about any training or information you have received since the initial surgery.

____

____

____

____

____

Q2.3 Who have you received training or information from? [select all that apply]

- ☐ Hospital team (1)
- ☐ Community Children's Nurse (CCN) (2)
- ☐ Other [please specify] (3) ____

Q2.4 What type of training/teaching have you received? [select all that apply]

- ☐ Given information verbally by a healthcare professional (23)
- ☐ Demonstrations by a healthcare professional (24)
- ☐ Practised on my child supervised by a healthcare professional (32)
- ☐ Demonstrations by another parent or family member (39)
- ☐ Given a written booklet (33)
- ☐ Directed to a website for information (34)
- ☐ Simulation practice (e.g. practised with a doll or some equipment) (35)
- ☐ Videos (38)
- ☐ Other [Please state] (37) ____

Q2.5 What was good about the training you received to care for your child's gastrostomy?

____

____

____

____

____

Q2.6 What could have been improved about your training or was there anything missed?

____

____

____

____

____

Q2.8 How confident did you feel to care for your child's gastrostomy **in the first week** at home after their surgery?

- Not at all confident (1)
- Slightly confident (2)
- Moderately confident (3)
- Mostly confident (4)
- Fully confident (5)

Q2.9 What were you not confident with?

____

____

____

____

____

Experiences of ongoing support

Section 3 of 5:
In this section we would like to understand how confident you feel now and what ongoing support you have found helpful or might want.

Q3.1 How confident do you feel **now** caring for your child's gastrostomy?

- ○ Not at all confident (1)
- ○ Slightly confident (2)
- ○ Moderately confident (3)
- ○ Mostly confident (4)
- ○ Fully confident (5)

Q3.2 Would it be helpful to receive any further training or support?

- ○ Definitely yes (1)
- ○ Probably yes (2)
- ○ Probably not (3)
- ○ Definitely not (4)

Q3.3 If yes, what would you like further training or support with?

____

____

____

____

____

Page B, *et al. BMJ Paediatrics Open* 2022; 5:e001068. doi: 10.1136/bmjpo-2021-001068

Q3.4 How helpful do you find the following sources for ongoing information and support? (Please select all that apply)

	Very helpful	Quite helpful	Not very helpful	Not applicable
Contacting your community nurse	○	○	○	○
Contacting your hospital team (e.g. specialist nurse)	○	○	○	○
Facebook groups	○	○	○	○
Conversations with other parents of children with similar needs	○	○	○	○
Written information/booklets provided by a healthcare professional	○	○	○	○
NHS websites	○	○	○	○
Charities	○	○	○	○
Videos	○	○	○	○
Other [please state]	○	○	○	○

Section 4 of 5: *We are working to develop training and resources for parents new to gastrostomy care.* In this section we would like to get your views on what training and support is most helpful to parents generally.

Q4.1 Which formats for training do you think are most useful?

	Extremely useful	Very useful	Moderately useful	Slightly useful	Not at all useful
Demonstrations by healthcare professionals	○	○	○	○	○
Practice supervised by a healthcare professional	○	○	○	○	○
Written booklets	○	○	○	○	○
Videos	○	○	○	○	○
Simulation training (e.g. hands on practice with a doll or some equipment)	○	○	○	○	○
Online training	○	○	○	○	○
Group training sessions with other parents	○	○	○	○	○
Training by an experienced parent	○	○	○	○	○

Q4.2 If you use Facebook groups for ongoing support relating to your child's gastrostomy, are there any you would recommend to other parents? If yes, please name.

____

____

____

____

____

Q4.3 We know that some children and parents find some tasks distressing, such as changing a gastrostomy button. Do you have tips to share with parents, or know of any resources that might be helpful to other parents?

____

____

____

____

____

Section 5 of 5: *We are developing some training videos for parents who are new to gastrostomy care. Some of the videos may also be helpful to more experienced parents and for training staff. In this final section we would like to get your views on what these videos should look like.*

Q5.1 We have started creating some videos. Here is an example video:

Q5.2 What did you like about the video?

Q5.3 What could be improved or was missing in the video?

Page B, *et al. BMJ Paediatrics Open* 2022; 5:e001068. doi: 10.1136/bmjpo-2021-001068

Q5.4 Which of the following videos do you think would be helpful to other parents?

	Really helpful	Quite helpful	Not very helpful	Not applicable to me
Caring for the stoma/gastrostomy site	○	○	○	○
Flushing the tube	○	○	○	○
Giving a feed	○	○	○	○
Giving medications	○	○	○	○
Venting (letting air or gas out)	○	○	○	○
Changing the water in the balloon of the gastrostomy button	○	○	○	○
How to change a gastrostomy button	○	○	○	○
What to do if the tube blocks	○	○	○	○
What to do if the tube comes out	○	○	○	○

Q5.5 Some of the videos will cover the technical aspects of care. We also recognise that there are wider topics which parents might find helpful. Which of the suggestions below might be helpful?

	Really helpful	Quite helpful	Not very helpful
Recommendations for Facebook groups to join	○	○	○
Tips for managing your child's distress, e.g. when changing a button	○	○	○
Advice for new parents from more experienced parents	○	○	○

Q5.6 Do you have any other suggestions for topics or things we should consider?

____

____

____

____

____

Q5.7 Where should the videos be filmed?

○ Home

○ Hospital

○ Mixture

Q5.8 Who should be delivering the teaching/information in the videos?

- ◯ Parents
- ◯ Healthcare professionals
- ◯ Mixture

Q5.9 Do you have any final comments for us on videos or other resources to support parents?

____

____

____

____

____

Thank you very much for your time today. Your thoughts and advice will be really helpful to us as we develop resources for preparing and supporting families.

Do you have any final comments for us?

____

____

____

____

____

As a thank you for your help we will be sending all participants who complete the full survey a voucher (£10 M&S e-giftcard). Please leave your email address here so we can send it to you (we will aim to email it to you within two weeks).

____

Would you like to be emailed a summary of the findings from this project?

○ Yes

○ No

We are currently developing more videos for families new to gastrostomy care. The videos will also be used for training staff. We will need help from parents later in the year to rate/evaluate the videos and resources.

Would you be willing to do this? We will compensate you for your time.

○ Yes, please contact me to help rate/evaluate more videos

○ No, please don't contact me about this.

Below is a link to a list of charities and resources which you may find helpful as a source of information and support. If this survey has raised any questions or concerns about the care of your child, please contact a healthcare professional for advice: Charities and other resources

Further details about the survey

For all the closed-ended/quantitative questions we used qualtrics' 'request response' option. This function rem was unanswered questions on the page before they could move on to the next page, but it did not force partici ended qualitative questions did not request a response. We included participants who completed all the quanti
The back button was enabled so participants could go back and check their answers.
We did not do any formal checks for unique site visitors. We identified one participant who had completed the email addresses submitted for the vouchers. The participants' second response was excluded.
