# Supplementary material for: Training and support for caring for a child’s gastrostomy: a survey with family carers
Source: BMJ Paediatr Open. 2021 Jul 27;5(1):e001068. doi: 10.1136/bmjpo-2021-001068 (PMC8317118; doi:10.1136/bmjpo-2021-001068)
Supplement: Supplementary data [file bmjpo-2021-001068supp002.pdf]

Supplementary File 2: Family carers' recommendations for topics for videos

Participants were asked to rate various topics for videos on a three point scale with 1 indicating 'not very helpful' and 3 indicating 'very helpful'. The vast majority of topics were rated as 'very helpful', with the lowest mean score being 2.45. Topics related to problem solving were generally rated as slightly more helpful than routine care.

Table 1 Participants' rating of which topics would be most helpful to cover in videos

	M (SD)	Very helpful	Quite helpful	Not very helpful	Not applicable
What to do if the tube comes out	2.90 (0.3)	129 (88%)	13 (9%)	1 (1%)	3 (2%)
What to do if the tube blocks	2.86 (0.4)	126 (86%)	17 (12%)	1 (1%)	2 (1%)
How to change a gastrostomy button	2.84 (0.4)	118 (81%)	18 (12%)	2 (1%)	8 (5%)
Venting (letting air or gas out)	2.82 (0.4)	114 (78%)	23 (16%)	1 (1%)	8 (5%)
Changing the water in the balloon of the gastrostomy button	2.79 (0.4)	110 (75%)	27 (18%)	1 (1%)	8 (5%)
Advice for new parents from more experienced parents	2.76 (0.5)	115 (79%)	27 (18%)	4 (3%)	0 (0%)
Caring for the stoma/gastrostomy site	2.72 (0.5)	106 (73%)	37 (25%)	1 (1%)	2 (1%)
Tips for managing child's distress (e.g. when changing button)	2.63 (0.6)	100 (68%)	37 (25%)	8 (5%)	1 (1%)
Giving a feed	2.63 (0.5)	94 (64%)	48 (33%)	3 (2%)	1 (1%)
Giving medications	2.62 (0.5)	93 (64%)	50 (34%)	3 (2%)	0 (0%)
Recommendations of Facebook groups to join for support	2.49 (0.6)	82 (56%)	52 (36%)	11 (8%)	1 (1%)
Flushing the tube	2.45 (0.6)	76 (52%)	57 (39%)	11 (7.6%)	2 (1%)

Family carers' suggestions of other topics to include

Family carers had various suggestions for other topics for videos. Common technical topics included blended diet, tips for specific problems (e.g. replacing connector end of Freka peg) and different types of feeding tubes. They also had suggestions relating to wider support needs, including advice on doing daily activities, ways of explaining feeding tubes to children and siblings and reassuring other families that children can still do normal activities: "*Showing people that they can get out and about with their child still. I know parents who return home to feed their child it would be nice to show how a gtube doesn't stop fun*."
