# Supplementary material for: Training and support for caring for a child’s gastrostomy: a survey with family carers
Source: BMJ Paediatr Open. 2021 Jul 27;5(1):e001068. doi: 10.1136/bmjpo-2021-001068 (PMC8317118; doi:10.1136/bmjpo-2021-001068)
Supplement: Author's manuscript [file bmjpo-2021-001068.draft_revisions.pdf]

BMJ Paediatrics Open

BMJ Paediatrics Open is committed to open peer review. As part of this commitment we make the peer review history of every article we publish publicly available.

When an article is published we post the peer reviewers' comments and the authors' responses online. We also post the versions of the paper that were used during peer review. These are the versions that the peer review comments apply to.

The versions of the paper that follow are the versions that were submitted during the peer review process. They are not the versions of record or the final published versions. They should not be cited or distributed as the published version of this manuscript.

BMJ Paediatrics Open is an open access journal and the full, final, typeset and author-corrected version of record of the manuscript is available on our site with no access controls, subscription charges or pay-per-view fees (http://bmjpaedsopen.bmj.com).

If you have any questions on BMJ Paediatrics Open's open peer review process please email info.bmjpo@bmj.com

BMJ Paediatrics Open

Training and supporting parents to care for their child's gastrostomy: Can we do better?

Journal:	*BMJ Paediatrics Open*
Manuscript ID	bmjpo-2021-001068
Article Type:	Original research
Date Submitted by the Author:	16-Feb-2021
Complete List of Authors:	Page, Bethan; University of Oxford, Experimental Psychology<br>Butler, Siobhan; Helen and Douglas House<br>Smith, Colette; Oxford Health NHS Foundation Trust<br>Lee, Alex CH; Oxford University Hospitals NHS Foundation Trust<br>Vincent, Charles A; University of Oxford, Experimental Psychology
Keywords:	Gastroenterology, Health services research, Nursing

SCHOLARONE™
Manuscripts

I, the Submitting Author has the right to grant and does grant on behalf of all authors of the Work (as defined in the below author licence), an exclusive licence and/or a non-exclusive licence for contributions from authors who are: i) UK Crown employees; ii) where BMJ has agreed a CC-BY licence shall apply, and/or iii) in accordance with the terms applicable for US Federal Government officers or employees acting as part of their official duties; on a worldwide, perpetual, irrevocable, royalty-free basis to BMJ Publishing Group Ltd ("BMJ") its licensees and where the relevant Journal is co-owned by BMJ to the co-owners of the Journal, to publish the Work in this journal and any other BMJ products and to exploit all rights, as set out in our licence.

The Submitting Author accepts and understands that any supply made under these terms is made by BMJ to the Submitting Author unless you are acting as an employee on behalf of your employer or a postgraduate student of an affiliated institution which is paying any applicable article publishing charge ("APC") for Open Access articles. Where the Submitting Author wishes to make the Work available on an Open Access basis (and intends to pay the relevant APC), the terms of reuse of such Open Access shall be governed by a Creative Commons licence – details of these licences and which Creative Commons licence will apply to this Work are set out in our licence referred to above.

Other than as permitted in any relevant BMJ Author's Self Archiving Policies, I confirm this Work has not been accepted for publication elsewhere, is not being considered for publication elsewhere and does not duplicate material already published. I confirm all authors consent to publication of this Work and authorise the granting of this licence.

Confidential: For Review Only

Training and supporting parents to care for their child's gastrostomy: Can we do better?

Bethan Page[1], Siobhan Butler[2], Colette Smith[3] Alex CH Lee[4], Charles A Vincent[1]

1 Department of Experimental Psychology, New Radcliffe House, Radcliffe Observatory Quarter, Woodstock Road, Oxford, OX2 6AE, UK

2 Helen & Douglas House, Oxford, UK

3 Oxford Health NHS Foundation Trust, UK

4 Oxford University Hospitals NHS Foundation Trust, UK

Correspondence to

Bethan Page, Department of Experimental Psychology, University of Oxford, New Radcliffe House, Radcliffe Observatory Quarter, Oxford, UK, OX2 6GG, Bethan.page@psy.ox.ac.uk

WORD COUNT: 2496

Confidential: For Review Only

ABSTRACT

Objective

The aim of this study was to explore parents' experiences of training and ongoing support for caring for their child's gastrostomy, and to get their views on how this could be improved.

Methods

A mixed-methods online survey with 147 families who care for a child with a gastrostomy. Parents rated their own experience of training and support and made recommendations for how training and support could be improved for future parents.

Results

Two in five parents rated their confidence caring for their child's gastrostomy as very low in the first few weeks after surgery. Many parents felt that demonstrations in hospital were too brief. The quality of support parents received in the community varied. Parents benefitted from ongoing learning and support from other parents. Videos and simulation practice were rated as useful, in addition to face-to-face supervised practice with a clinician. Parents liked how real life the example video shown was, and rated nearly all suggested video topics as "very helpful", especially troubleshooting topics.

Conclusions

The training parents receive to care for their child's gastrostomy varies considerably. Many parents felt that their training was too basic or brief, and did not prepare them for managing problems. Improved training and support, e.g. through instructional videos, could make a substantial difference to parents' experiences, reduce the risk of harm to children and reduce demand on NHS services. Training needs to begin prior to the child's surgery and be regarded as an essential component of the care package.

Confidential: For Review Only

What is already known on this topic

- It is now commonplace for parents to carry out complex medical procedures for children at home.
- Some studies have reported safety concerns around the practices of parents caring for children with gastrostomies

What this study adds

- Training for parents is variable, and sometimes poor, leaving many parents feeling anxious and under-confident.
- Parents valued supervised practice with healthcare professionals, videos featuring parents and clinicians and more hands on practice, including using models/dolls.
- Training in a short hospital admission is often insufficient to prepare parents.
- Training should begin prior to the hospital admission for surgery and be regarded as an essential component of the care package.

Confidential: For Review Only

INTRODUCTION

Many medical procedures, which were once carried out only by healthcare professionals in hospital wards, are now performed by families at home.[1-3] There are increasing numbers of children with complex medical needs.[4,5] Their parents perform a range of medical procedures for them at home, including feeding tube care, home oxygen, tracheostomy care and bowel washouts.[1,4,6] There are numerous benefits for families and the healthcare system when children can be cared for at home, rather than in healthcare settings,[7,8] but there are also substantial risks.[2] An analysis of incident reports on enteral tube feeding identified training and support for parents as a weakness in the system.[9] Without consistently high-quality training and support for parents, children are at risk of harm, and parents feel under-confident, anxious and alone.[6,10]

Caring for a child with a gastrostomy

A gastrostomy is a surgically placed device where feeds are delivered directly into the stomach. Gastrostomies are common in children with severe chronic illnesses and neurodisability who have difficulty swallowing or cannot get adequate nutrition through eating and drinking.[11,12] Parents have to learn to administer feeds, water and medications via the tube, to clean and care for the stoma site, and to manage complications such as leakage and blocked tubes. Parents may also learn to change a gastrostomy button. Parents normally receive some bedside teaching in hospital (during a hospital stay of around 2 days), with ongoing support from community nurses, but this may not be sufficient to ensure safe care at home. There are some safety concerns reported in the literature around the practices of parents caring for children with gastrostomies.[9,13,14] In a recent study on same-day discharge for gastrostomy surgery, the most common reason for a delayed discharge was parents not feeling sufficiently confident caring for the gastrostomy.[15] Improving training and support for families could reduce callouts to over-stretched community teams, as well as preventing avoidable Emergency Department visits and admissions, e.g. through appropriate timely management of a dislodged button.

Many parents caring for children with complex medical needs develop substantial expertise over time.[6,9,10] However training for parents can be informal and highly variable. [6,10,11] Good quality training is key for optimising outcomes for children.[16] The aim of this study is to explore parents' experiences of training and ongoing support for caring for their child's gastrostomy, and to find out their views on how training and support could be improved. This paper is a part of a longer-term project to develop a package of training and support for families.

Confidential: For Review Only

METHODS

Survey aims

The first aim was to understand parents' experiences of training and support for caring for their child's gastrostomy. The second aim was to understand parents' recommendations for improving training and support.

Survey development

The survey was developed in stages. In the initial exploratory stage, we reviewed the literature on gastrostomy training and consulted parent representatives, nurses and paediatricians from the community and hospital. We carried out a preliminary survey with 50 families who performed a range of medical procedures, focusing on their experiences of training, support and the emotional demands. The survey was primarily qualitative. Box 1 highlights some of the key themes from this preliminary survey which informed the design of the present study.

The survey reported here focused specifically on gastrostomy care. The survey instrument was drafted, piloted and revised in consultation with parent representatives, children's nurses and paediatricians from the community, a paediatric gastrointestinal surgeon and a specialist surgical nurse. The team were asked to comment on the suitability of the questions, readability and length. Recruitment strategies were developed based on advice from our parent representatives, clinicians and charities.

Confidential: For Review Only

Box 1: Summary of key themes from preliminary survey

Parents feeling scared and unprepared

- In the first few weeks of providing care at home, the overwhelming majority of parents were scared, ranging from *"a little worried" to "terrified, out of my depth."* Some parents talked about feeling isolated and exhausted. A few commented on the fear of getting something wrong: *"Terrified we would do it wrong, we weren't really sure what was right or wrong."*

Variability in experiences of training

- Some parents described very thorough training and some very limited training. Few felt their training fully prepared them for caring for their child at home. For example one parent reflected on the limitations of training in the hospital for preparing you to care for a child in the home environment: *"Real life situations are very different than in a classroom or hospital setting - you may not have a clean surface to lay things out on or they may be sitting up and not able to lie them down for instance."*

The emotional demands on parents

- Tasks which children find distressing or painful were commonly mentioned as being difficult, such as passing a nasogastric tube or changing a gastrostomy button: *"I find it really difficult to carry out these tasks when I'm the parent and having to inflict these procedures on my child which may cause pain/upset."*

Survey design and content

The survey was a fully mixed-methods survey with the qualitative data intended to complement, illustrate and expand the quantitative data.[17] The survey tool used was Qualtrics. The survey is available in Supplementary File 1.

Participants

Participants were recruited through UK charities and local charities and through our parent-representatives who posted on Facebook parent support groups. Some parents also choose to share the survey with friends/family. The inclusion criteria was any parent or family carer who provides gastrostomy care to a child or young person at home. By family carer, we included any unpaid carer (parent, relative, friend) who actively participates in caring for a child with a gastrostomy. To take part family carers needed to be at least 18 years old. Participants received a £10 voucher for taking part. All participants gave informed consent before taking part and consented for their data to be used in publications (The Medical Sciences Inter-Divisional Research Ethics Committee, Oxford

For peer review only

University, R56623/RE004). The data was collected between July and September 2020. We aimed to recruit at least 100 families to capture a broad range of experiences, including families new to gastrostomy care and some more experienced.

Analysis

Descriptive statistics were computed for all close-ended questions, using SPSS Statistics 25. Participants who did not complete the full survey were excluded. The open-ended questions were coded in NVivo 12 using inductive content analysis, to group responses based on surface level of meaning.[18] Answers were coded line by line, and grouped into categories emerging from the data. These were summarised in the text and illustrated with quotes from participants.

Patient and Public Involvement

Two parent representatives were involved in the design, conduct and dissemination of the research.

RESULTS

Participants

One-hundred-and-forty-six participants completed the full survey, and 195 participants started the survey. The majority were mothers (91%), there was a range of ages of the children and time since initial gastrostomy surgery. The most common types of devices were gastrostomy buttons and percutaneous endoscopic gastrostomy (PEG) tubes. Table 1 gives more detail about the families.

Table 1 About the families who completed the full survey

		N (%)
**Relation to child**		
	Mother	133 (91%)
	Father	8 (6%)
	Other family member	5 (3%)
**Age of children (years)**		
	0-4	50 (34%)
	5-9	38 (26%)
	10-14	39 (27%)
	15-20	19 (13%)
**Time since initial gastrostomy surgery (years)**		
	<1 year	41 (28%)
	1-2	27 (18%)
	3-4	27 (18%)

Confidential: For Review Only

5+ years	63 (43%)
**Type of gastrostomy device child has or previously had**	
PEG tube	73 (50%)
Gastrostomy button (MINI or Mic-Key)	115 (79%)
Another device (e.g. gastro-jejunostomy (GJ) tube)	23 (16%)

Parents' experiences of initial training

Parents' experiences of training were hugely variable (see Box 2). Most parents described receiving some training in hospital but the nature and extent varied considerably. Descriptions of training as "*brief*" or "*basic*" were common. Some parents felt unprepared and anxious: *"It was scary because we were worried about it getting caught/pulled and hurting or causing damage to our little boy. We didn't get much practice before being left to do it on our own so you are triple checking yourself and worrying did I do it right."* Some parents described receiving further training at home by community nurses or representatives from a feeding company. Parents described learning through other parents, often through Facebook groups: *"Also had amazing advice from other mums whose children have tubes who I found via Facebook groups - game changing stuff."*

Confidential: For Review Only

Box 2 Example quotes illustrating the variability of training

Training sometimes brief & basic:

- *"A brief 5 mins of basics, a leaflet and home. A few weeks later a Nutricia nurse came to the house to show us the pump."*
- *"We were admitted for the surgery and spent two further days in the ward after, received very basic training on the use of the peg and left feeling absolutely terrified about using it!"*
- *"Need much more training than 10 minutes in consultant's office!"*

Primarily self-taught with support from other parents:

- *"The official training - nothing good. Absolutely disastrous, vague, unsupportive. The unofficial training and the info I sought out for myself - clear, helpful videos from other parents, useful approach of gastrostomy nurse. Properly child-centric, helpful stuff."*

More community support needed:

- *"The community nursing team rely too much on the surgical nurse to do the training and then they just catch up with a chat following any training given at the bedside. This training is not detailed/long enough for those dealing with such a complex medical needs child."*

Thorough training and support received:

- *"Our daughter was in hospital long terms due to a range of factors. We were able to observe nurses undertaking feeds, using pump, giving medication. We were also given an information pack and work book to go through, and we were observed by nurses until confident and competent to do ourselves. We were given support in community… with regards to changing button, taking care of button, and annual refresher also. Received updates with regards to how much water to inflate balloon with, frequency of Ph testing etc."*

The majority of parents reported receiving some training from their hospital team (n=115, 79%). A slightly smaller proportion received training from a Community Children's Nurse (CCN) (n=105, 72%). 35 participants (24%) mentioned training from another provider, most commonly a feeding company

(e.g. Nutricia). 82 (56%) families had received training from both the hospital and CCN team, and 8 (5%) reported not receiving training from either the hospital team or a CCN team.

Table 2 shows the types of training parents received. The most common types of training were verbal information, demonstrations and supervised practice from a healthcare professional. Very few participants received any simulation practice (hands on practice with a doll/equipment), or watched instructional videos. A few of the parents that did receive simulation practice commented on the usefulness of this: *"Really useful being able to have a little play with a tube and practice using the clip etc. before having to do it for real."*

Table 2 Types of teaching/training received by parents

**Types of teaching/training received**	**N (%)**
Given information verbally by a healthcare professional	129 (88%)
Demonstrations by a healthcare professional	125 (86%)
Practised on my child supervised by a healthcare professional	113 (77%)
Given a written booklet	85 (58%)
Simulation practice (practiced with a doll or some equipment)	19 (13%)
Directed to a website for information	13 (9%)
Demonstrations by another family member	7 (5%)
Other	5 (3%)
Videos	3 (2%)

Parents were asked how confident they felt caring for their child's gastrostomy in the first few weeks after surgery: 24 (16%) were not at all confident, 32 (22%) were slightly confident, 30 (21%) were moderately confident, 42 (29%) were mostly confident and 18 (12%) of parents said they were fully confident. At the time of the survey majority (n=117, 80%) said they felt fully confident caring for their child's gastrostomy (n=117, 80%). The most common concern parents had were around hurting their child, caring for site after surgery, knowing "*what was normal*" in relation to the stoma site healing, worries about the tube coming out and problems such as blocked tubes or granulation: *"The first time I experienced this [granulomas] I thought my sons intestines were coming out! Nobody had ever mentioned it to me nor had I ever seen anything like it!"*

Ongoing support and training

Parents were asked which sources were most helpful for ongoing support (see Table 3). Contacting your community nurses was rated as "very helpful" by a majority, with less than a quarter rating

Confidential: For Review Only

hospital teams as a very helpful source of support. Notably conversations with other parents was rated as "very helpful" by 56%, with a further 32% ratings support from parents as "quite helpful". Parents in the survey offered advice for other parents, including tips for managing their child's distress when changing a gastrostomy button.

Table 3 Sources of ongoing support: how helpful are they?

	Very helpful	Quite helpful	Not very helpful	Not applicable
Contacting your community nurse	90 (62%)	28 (19%)	13 (9%)	15 (10%)
Contacting your hospital team	34 (23%)	37 (25%)	38 (26%)	37 (25%)
Facebook groups	54 (37%)	67 (46%)	11 (8%)	14 (10%)
Conversations with other parents of children with similar needs	82 (56%)	47 (32%)	3 (2%)	14 (10%)
Written information booklets provided by a healthcare professional	28 (19%)	68 (47%)	32 (22%)	18 (12%)
NHS websites	15 (10%)	55 (38%)	52 (36%)	24 (16%)
Charities	25 (17%)	53 (36%)	19 (13%)	49 (34%)
Videos	29 (20%)	50 (34%)	17 (12%)	50 (34%)

Even experienced parent carers considered that some further training might be helpful, with 14 (10%) saying definitely yes to further training or support, 29 (20%) probably yes, 81 (55%) probably not and 21 (14%) definitely not. The most common request was help with managing problems. A number of parents wanted refresher training, or updates on the latest guidance: "*making sure bad habits have not crept in and that we are up-to-date with any changes in how things are done."*

Parents' recommendations for improving training

Participants were asked which formats of training might be useful to other parents facing the challenge of caring for a child with a gastrostomy (see Table 4). Around three-quarters rated demonstrations and practice on my child supervised by a healthcare professional as extremely useful. Around two-thirds of parents felt that videos and simulation practice would be extremely or very useful. Parents felt that these additional forms of training would be a good addition to face-to-face training with a professional, but not a substitute: *"I think that the videos are good resources that parents can go back to however I think that face to face training is really important initially."*

Table 4 Which types of training might be helpful to other parents

	Extremely useful	Very useful	Moderately useful	Slightly useful	Not at all useful

Confidential: For Review Only

Demonstrations by a healthcare professional	105 (72%)	33 (23%)	6 (4%)	2 (1%)	0 (0%)
Practised on my child supervised by a healthcare professional	110 (75%)	29 (20%)	5 (3%)	1 (1%)	1 (1%)
Written booklets	32 (22%)	39 (27%)	51 (35%)	19 (13%)	5 (3%)
Videos	40 (27%)	58 (40%)	29 (20%)	14 (10%)	5 (3%)
Simulation training (practicing with a doll or some equipment)	47 (32%)	45 (31%)	36 (25%)	10 (7%)	7 (5%)
Online training	18 (12%)	33 (23%)	47 (32%)	30 (21%)	17 (12%)
Group training with other parents	20 (14%)	34 (23%)	39 (27%)	31 (21%)	21 (14%)
Training by an experienced parents	15 (10%)	16 (11%)	32 (22%)	51 (35%)	32 (22%)

Parents had many suggestions for improving training. A common suggestion was more training and information about common problems including what to do if the button comes out, and requests for specific types of training, such as more hands on training. Some parents commented on the timing of training as they struggled to take information on board while in hospital: *"I think we should have had more [training] before his surgery and not while he was in theatre as we were so anxious I'm not sure how much we took in or how valuable doing it then was."* A few parents said they would like to have been put in touch with other families: *"Opportunity to do group talks or training would be good. Nice way to meet other families and build a support network."*

Parents' recommendations for video development

Parents were shown a sample video showing a parent administering a bolus of water to their child. Parents liked how "real life" the video was: the child was wriggling during the procedure, it was done at home, it was relaxed and the mother was talking to the child throughout. One parent commented that: *"I've hated the teaching videos previously as they seem rather clinical but this was fantastic."* When asked where training videos should be filmed, 54 (37%) said at home, 4 (3%) said in hospital, and 88 (60%) said a mixture of both locations. 116 (80%) of participants wanted both healthcare professionals and parents to feature, 12 (18%) wanted just healthcare professionals and 8 (12%) wanted just parents. Parents rated different topics for videos: the vast majority were rated as 'very helpful' (see Supplementary file 2).

https://mc.manuscriptcentral.com/bmjpo

Confidential: For Review Only

DISCUSSION

The training parents receive to care for their child's gastrostomy varies considerably, and is sometimes of poor quality. Parents are often anxious in the first few weeks of caring for their child at home and concerned about doing something wrong or hurting their child. Demonstrations in hospital were frequently described as too brief, and ongoing support in the community was highly variable. Parents valued support from other parents. Videos and simulation practice were rated as useful preparation, in addition to face-to-face supervised practice with a healthcare professional. Parents wanted videos to feature parents and health professionals and for at least some of the videos to be filmed at home.

Implications for the design of services

Shorter hospital stays are increasingly advocated for gastrostomy surgery, including same day discharges,[15] meaning that there is little time to train parents during admission. Preparation and training therefore needs to begin before admission, with time during or after admission for parents to practice on their child supervised by designated professionals. Parents need to be prepared for caring for their child's gastrostomy supported by either hospital or community nurses before surgery takes place. Instructional videos and written materials should be available to all families awaiting gastrostomy surgery, to be revisited as needed. To enable more opportunity for hands-on practice, parents could practice with dolls and equipment at home: there is substantial evidence on the benefits of repeated hand-on-practice in the medical education literature.[19,20] There is potential to make more use of online training and support, including group video-calls for families. Training resources should be co-designed with families and address their emotional needs as well as the technical aspects of care. Box 3 gives an overview of practical suggestions for improving training. We are currently creating a library of videos co-produced with families, paediatricians and nurses from the hospital and community and piloting hands-on practice with equipment and 3D-printed models (see Figure 1).

Confidential: For Review Only

Box 3: Practical suggestions for improving training

Before surgery

- Parents watch videos teaching them about routine care of a gastrostomy, how to manage common problems, and advice and tips from more experienced families
- Parents receive a home visit from community nurse for face-to-face teaching with models and to ask questions
- Parents repeatedly practice with a doll/equipment to become familiar with the basics before their child's surgery, e.g. connecting extension tube
- Parents invited to a group call with other families awaiting surgery to ask questions to surgical team and meet other parents
- Parents directed to Facebook groups and other peer support options

During hospital admission

- Supervised practice doing procedures on child after surgery with support of designated nurse

After hospital admission

- Community nurses provide further support and teaching through home visits and video-calls
- Parents can revisit videos and written materials as needed including videos on managing common problems such as granulation
- Parents supported by community nurses to learn to change a button, through repeated practicing with models in addition to supervised changes on their child

Review Only

Strengths and limitations

The sample was recruited predominantly through charities and Facebook groups. This enabled us to recruit a large sample of families from across the country, to ensure experience of a wide range of clinical services. The survey included families new to gastrostomy care and very experienced families. It is difficult to know how representative the samples are of all families: we may have recruited families who are more engaged in their child's care or families who felt unprepared and sought help through Facebook groups and charities. We did not collect demographic data on the families so cannot tell the socio-economic, health literacy or ethnicity of families. Families were very engaged and many offered to help produce videos.

CONCLUSIONS

Training for parents to care for their child's gastrostomy is highly variable and sometimes of poor quality. Parents value face-to-face training with a healthcare professional, videos which show "real-life" featuring families and clinicians and hands-on practice including with dolls/equipment. There is much that can be done to support and train families which need not involve substantial staff time

and resources, and can improve the lives of families and reduce the risk of problems and complications which cause harm to children and increase demand on NHS resources. There is huge value in involving families in designing improvements to services, and treating them as equal partners in children's care.

Confidential: For Review Only

REFERENCES

1. Ten Haken I, Ben Allouch S, Van Harten WH. The use of advanced medical technologies at home: A systematic review of the literature. *BMC Public Health* 2018;**18**(1):284.

2. Institute for Healthcare Improvement. No Place Like Home: Advancing the Safety of Care in the Home 2018. Available from: http://www.ihi.org/resources/Pages/Publications/No-Place-Like-Home-Advancing-Safety-of-Care-in-the-Home.aspx

3. Reinhard S, Levine C, Samis S. Home alone: family caregivers providing complex care. *Gerontologist* 2017;**56**(Suppl_3):636–636.

4. Cohen E, Berry JG, Sanders L, Schor EL, Wise PH. Status Complexicus? The Emergence of Pediatric Complex Care. *Pediatrics* 2018;**141**(Suppl 3):S202–S211.

5. Fraser L, Gibson-Smith D, Jarvis S, Norman P, Parslow R. "Make Every Child Count" Estimating current and future prevalence of children and young people with life-limiting conditions in the United Kingdom. 2020. Available from: https://www.togetherforshortlives.org.uk/resource/make-every-child-count/

6. Page BF, Hinton L, Harrop E, Vincent C. The challenges of caring for children who require complex medical care at home: 'The go between for everyone is the parent and as the parent that's an awful lot of responsibility.' *Heal Expect* 2020;**23**(5):1144-1154.

7. Glasson EJ, Forbes D, Ravikumara M, Nagarajan L, Wilson A, Jacoby P, et al. Gastrostomy and quality of life in children with intellectual disability: A qualitative study. *Arch Dis Child* 2020, **105**(10):969-974.

8. Mitchell TK. The impact of medical technology upon the home and feelings of at-homeness for children and young people with complex health care needs and their family members. PhD Thesis Tracy Karen Mitchell, Edge Hill University. 2020. Available at: https://research.edgehill.ac.uk/en/studentTheses/the-impact-of-medical-technology-upon-the-home-and-feelings-of-at

9. Page B, Nawaz R, Haden S, Vincent C, Lee ACH. Paediatric enteral feeding at home: An analysis of patient safety incidents. *Arch Dis Child* 2019;**104**:1174-1180.

10. Spiers G, Beresford B. "It goes against the grain": A qualitative study of the experiences of parents' administering distressing health-care procedures for their child at home. *Heal Expect* 2017; **20**(5):920-928.

https://mc.manuscriptcentral.com/bmjpo

Confidential: For Review Only

11. Burdall OC, Howarth LJ, Sharrard A, Lee ACH. Paediatric enteral tube feeding. *Paediatrics and Child Health* 2017; **27**(8):P371-377,2017.

12. Puntis JWL. Benefits and management of gastrostomy. *Paediatrics and Child Health* 2009;**19**(9):415–24.

13. Evans S, Shelton F, Holden C, Daly A, Hopkins V, MacDonald A. Monitoring of home safety issues in children on enteral feeds with inherited metabolic disorders. *Arch Dis Child* 2010; **95**(9): 668–672.

14. Evans S, Preston F, Daly A, Ashmore C, MacDonald A, Holden C. Home enteral tube feeding in children with inherited metabolic disorders: a review of long-term carer knowledge and technique. *J Hum Nutr Diet* 2012;**25**(6):520-525.

15. Dekonenko C, Svetanoff WJ, Osuchukwu OO, Pierce AL, Orrick BA, Sayers KL, et al. Same-day discharge for pediatric laparoscopic gastrostomy. *J Pediatr Surg* 2020; **56**(1):26-29.

16. Berman L, Hronek C, Raval M V, Browne ML, Snyder CL, Heiss KF, et al. Pediatric gastrostomy tube placement: Lessons learned from high-performing institutions through structured Interviews. *Pediatr Qual Saf* 2017;**2**(2):e016.

17. Terry G, Braun V. Short but Often Sweet. In: Collecting Qualitative Data. Cambridge University Press; 2017.

18. Elo S, Kyngäs H. The qualitative content analysis process. *J Adv Nurs* 2008;**62**(1):107–15.

19. Motola I, Devine LA, Chung HS, Sullivan JE, Issenberg SB. Simulation in healthcare education: A best evidence practical guide. AMEE Guide No. 82. *Med Teach* 2013;**35**(10):142–59.

20. McGaghie WC. Does Simulation-based Medical Education with Deliberate Practice Yield Better Results than Traditional Clinical Education? A Meta-Analytic Comparative Review of the Evidence. *Acad Med* 2012;**86**(6):706–11.

Confidential: For Review Only

Acknowledgements

We would like to thank all the parents who completed the survey. We are grateful for the expertise of our multidisciplinary stakeholder group, which includes parents, paediatricians, children's nurses and specialist surgical nurses. We thank the team for their ongoing work developing training for parents. This work was supported a grant from Health Foundation's Q Exchange Programme. BP is supported by the Economic and Social Research Council [grant number ES/J500112/1]. CV is supported by the Health Foundation.

Contributorship statement

BP and CV conceived and designed the study. The survey was designed and revised by BP with support from SB, CS, AL & CV. BP analysed the data. BP drafted the manuscript with support from SB, CS and CV. All authors provided critical feedback on the manuscript and analysis. All authors approved the final manuscript.

Competing interests

None declared.

Data sharing statement

Data may be obtained upon reasonably request by contacting the corresponding author. The survey tool used is available in the supplementary information.

Confidential: For Review Only

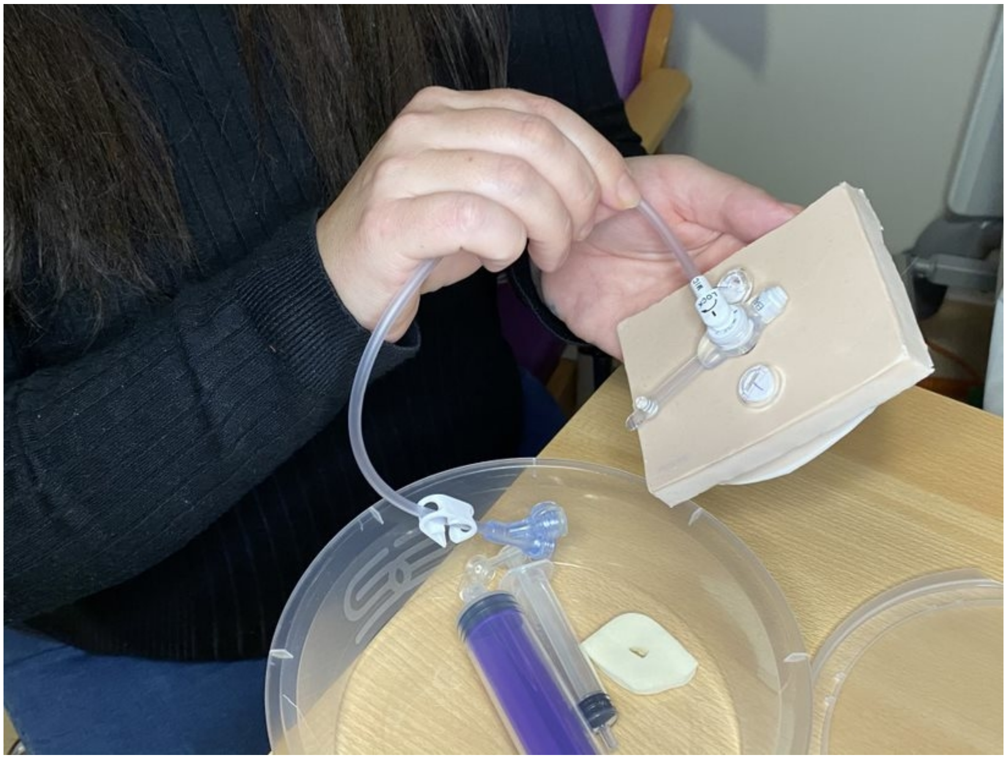

69x51mm (300 x 300 DPI)

Supplementary File 1: Survey Tool

Overview of survey

Gastrostomy care: an online survey for parents on training and support needs

- This survey is for family carers (e.g. mums, dads, grandparents) who care for a child or young person who has a gastrostomy (e.g. PEG or button).
- This survey is part of a project to develop resources to better prepare and support families who are new to gastrostomy care. The project is a collaboration between researchers, parents and healthcare professionals.
- The findings will be shared widely with parents, charities, healthcare providers and researchers.

What do I have to do to take part?

- This study is an online survey and will take 15-20 minutes to complete.
- You will be asked about your experiences of learning to care for your child's gastrostomy, your experiences of ongoing support and what videos and resources might be helpful.
- To take part, the child you care for needs to be under 18 years and you need to be at least 18 years old.
- Please read the participant information sheet on the next page if you are interested in taking part.

Thank you for your time.

Page Break

Confidential: For Review Only

About you and your child

Section 1 of 5:
In this first section we would like to ask some questions about you and the child or young person you provide care for.

Q1.1 What is your relationship to the child that you care for?

◯ Mother (1)

◯ Father (2)

◯ Other [please specify] (4) ____

Q1.2 How old is your child?

____

Q1.3 How long ago did your child first have gastrostomy surgery?

◯ Less than a year (1)

◯ 1 - 2 years (2)

◯ 3 - 4 years (3)

◯ 5 + years (4)

Confidential: For Review Only

Q1.4 Which types of gastrostomy tube has your child had? [select all that apply]

- ☐ Gastrostomy button (e.g. MIC-KEY button, MINI button) (1)
- ☐ Percutaneous Endoscopic Gastrostomy (PEG) (e.g. Freka, Corflo) (2)
- ☐ Other [please specify] (3) ____

Experiences of learning and being trained

Section 2 of 5:
In this section we would like to understand what training and information you received to care for your child's gastrostomy and what you found helpful.

Parents have to learn to give feeds and medications, clean and care for the stoma site and learn what to do if there is a problem, such as if the PEG tube blocks or the gastrostomy button comes out. There are also other tasks you may have learnt to do such as changing a gastrostomy button.

Q2.1 Tell us about what training and information you received when your child first had gastrostomy surgery.

____

____

____

____

____

Q2.2 Tell us about any training or information you have received since the initial surgery.

____

____

Confidential: For Review Only

____

____

____

Q2.3 Who have you received training or information from? [select all that apply]

- ☐ Hospital team (1)
- ☐ Community Children's Nurse (CCN) (2)
- ☐ Other [please specify] (3) ____

Q2.4 What type of training/teaching have you received? [select all that apply]

- ☐ Given information verbally by a healthcare professional (23)
- ☐ Demonstrations by a healthcare professional (24)
- ☐ Practised on my child supervised by a healthcare professional (32)
- ☐ Demonstrations by another parent or family member (39)
- ☐ Given a written booklet (33)
- ☐ Directed to a website for information (34)
- ☐ Simulation practice (e.g. practised with a doll or some equipment) (35)
- ☐ Videos (38)
- ☐ Other [Please state] (37) ____

Confidential: For Review Only

Q2.5 What was good about the training you received to care for your child's gastrostomy?

____

____

____

____

____

Q2.6 What could have been improved about your training or was there anything missed?

____

____

____

____

____

Q2.8 How confident did you feel to care for your child's gastrostomy **in the first week** at home after their surgery?

◯ Not at all confident (1)

◯ Slightly confident (2)

◯ Moderately confident (3)

◯ Mostly confident (4)

◯ Fully confident (5)

Q2.9 What were you not confident with?

____

Confidential: For Review Only

Experiences of ongoing support

Section 3 of 5:
In this section we would like to understand how confident you feel now and what ongoing support you have found helpful or might want.

Q3.1 How confident do you feel **now** caring for your child's gastrostomy?

- ◯ Not at all confident (1)
- ◯ Slightly confident (2)
- ◯ Moderately confident (3)
- ◯ Mostly confident (4)
- ◯ Fully confident (5)

Q3.2 Would it be helpful to receive any further training or support?

- ◯ Definitely yes (1)
- ◯ Probably yes (2)
- ◯ Probably not (3)
- ◯ Definitely not (4)

Confidential: For Review Only

Q3.3 If yes, what would you like further training or support with?

Confidential: For Review Only

Q3.4 How helpful do you find the following sources for ongoing information and support? (Please select all that apply)

	Very helpful	Quite helpful	Not very helpful	Not applicable
Contacting your community nurse	○	○	○	○
Contacting your hospital team (e.g. specialist nurse)	○	○	○	○
Facebook groups	○	○	○	○
Conversations with other parents of children with similar needs	○	○	○	○
Written information/booklets provided by a healthcare professional	○	○	○	○
NHS websites	○	○	○	○
Charities	○	○	○	○
Videos	○	○	○	○
Other [please state]	○	○	○	○

Confidential: For Review Only

Section 4 of 5: *We are working to develop training and resources for parents new to gastrostomy care.* In this section we would like to get your views on what training and support is most helpful to parents generally.

Q4.1 Which formats for training do you think are most useful?

	Extremely useful	Very useful	Moderately useful	Slightly useful	Not at all useful
Demonstrations by healthcare professionals	○	○	○	○	○
Practice supervised by a healthcare professional	○	○	○	○	○
Written booklets	○	○	○	○	○
Videos	○	○	○	○	○
Simulation training (e.g. hands on practice with a doll or some equipment)	○	○	○	○	○
Online training	○	○	○	○	○
Group training sessions with other parents	○	○	○	○	○
Training by an experienced parent	○	○	○	○	○

Confidential: For Review Only

Q4.2 If you use Facebook groups for ongoing support relating to your child's gastrostomy, are there any you would recommend to other parents? If yes, please name.

Q4.3 We know that some children and parents find some tasks distressing, such as changing a gastrostomy button. Do you have tips to share with parents, or know of any resources that might be helpful to other parents?

Section 5 of 5: *We are developing some training videos for parents who are new to gastrostomy care. Some of the videos may also be helpful to more experienced parents and for training staff. In this final section we would like to get your views on what these videos should look like.*

Q5.1 We have started creating some videos. Here is an example video:

Confidential: For Review Only

Q5.2 What did you like about the video?

Q5.3 What could be improved or was missing in the video?

Confidential: For Review Only

Q5.4 Which of the following videos do you think would be helpful to other parents?

	Really helpful	Quite helpful	Not very helpful	Not applicable to me
Caring for the stoma/gastrostomy site	○	○	○	○
Flushing the tube	○	○	○	○
Giving a feed	○	○	○	○
Giving medications	○	○	○	○
Venting (letting air or gas out)	○	○	○	○
Changing the water in the balloon of the gastrostomy button	○	○	○	○
How to change a gastrostomy button	○	○	○	○
What to do if the tube blocks	○	○	○	○
What to do if the tube comes out	○	○	○	○

Confidential: For Review Only

Q5.5 Some of the videos will cover the technical aspects of care. We also recognise that there are wider topics which parents might find helpful. Which of the suggestions below might be helpful?

	Really helpful	Quite helpful	Not very helpful
Recommendations for Facebook groups to join	○	○	○
Tips for managing your child's distress, e.g. when changing a button	○	○	○
Advice for new parents from more experienced parents	○	○	○

Q5.6 Do you have any other suggestions for topics or things we should consider?

____

____

____

____

____

Q5.7 Where should the videos be filmed?

○ Home

○ Hospital

○ Mixture

Confidential: For Review Only

Q5.8 Who should be delivering the teaching/information in the videos?

- ◯ Parents
- ◯ Healthcare professionals
- ◯ Mixture

Q5.9 Do you have any final comments for us on videos or other resources to support parents?

____
____
____
____
____

Thank you very much for your time today. Your thoughts and advice will be really helpful to us as we develop resources for preparing and supporting families.

Do you have any final comments for us?

____
____
____
____
____

As a thank you for your help we will be sending all participants who complete the full survey a voucher (£10 M&S e-giftcard). Please leave your email address here so we can send it to you (we will aim to email it to you within two weeks).

____

Confidential: For Review Only

Would you like to be emailed a summary of the findings from this project?

- ◯ Yes
- ◯ No

We are currently developing more videos for families new to gastrostomy care. The videos will also be used for training staff. We will need help from parents later in the year to rate/evaluate the videos and resources.

Would you be willing to do this? We will compensate you for your time.

- ◯ Yes, please contact me to help rate/evaluate more videos
- ◯ No, please don't contact me about this.

Below is a link to a list of charities and resources which you may find helpful as a source of information and support. If this survey has raised any questions or concerns about the care of your child, please contact a healthcare professional for advice: Charities and other resources

Confidential: For Review Only

Supplementary File 2: Parents' recommendations for topics for videos

Parents were asked to rate various topics for videos on a three point scale with 1 indicating 'not very helpful' and 3 indicating 'very helpful'. The vast majority of topics were rated as 'very helpful', with the lowest mean score being 2.45. Topics related to problem solving were generally rated as slightly more helpful than routine care.

Confidential: For Review Only

Table 1 Parents rating of which topics would be most helpful to cover in videos

	Very helpful	Quite helpful	Not very helpful	Not applicable
What to do if the tube comes out	129 (88%)	13 (9%)	1 (1%)	3 (2%)
What to do if the tube blocks	126 (86%)	17 (12%)	1 (1%)	2 (1%)
How to change a gastrostomy button	118 (81%)	18 (12%)	2 (1%)	8 (5%)
Venting (letting air or gas out)	114 (78%)	23 (16%)	1 (1%)	8 (5%)
Changing the water in the balloon of the gastrostomy button	110 (75%)	27 (18%)	1 (1%)	8 (5%)
Advice for new parents from more experienced parents	115 (79%)	27 (18%)	4 (3%)	0 (0%)
Caring for the stoma/gastrostomy site	106 (73%)	37 (25%)	1 (1%)	2 (1%)
Tips for managing child's distress (e.g. when changing button)	100 (68%)	37 (25%)	8 (5%)	1 (1%)
Giving a feed	94 (64%)	48 (33%)	3 (2%)	1 (1%)
Giving medications	93 (64%)	50 (34%)	3 (2%)	0 (0%)
Recommendations of Facebook groups to join for support	82 (56%)	52 (36%)	11 (8%)	1 (1%)
Flushing the tube	76 (52%)	57 (39%)	11 (7.6%)	2 (1%)

Parents’ suggestions of other topics to include

Parents had a wide range of suggestions for other topics for videos. Common technical topics included blended diet, tips for specific problems (e.g. replacing connector end of Freka peg) and different types of feeding tubes. They also had suggestions relating to wider support needs, including advice on doing daily activities, ways of explaining feeding tubes to children and siblings and reassuring other parents that children can still do normal activities: “Showing people that they can get out and about with their child still. I know parents who return home to feed their child it would be nice to show how a gtube doesn't stop fun.”

Confidential: For Review Only

BMJ Paediatrics Open

Training and support for caring for a child's gastrostomy: A parental survey

Journal:	*BMJ Paediatrics Open*
Manuscript ID	bmjpo-2021-001068.R1
Article Type:	Original research
Date Submitted by the Author:	10-May-2021
Complete List of Authors:	Page, Bethan; University of Oxford, Experimental Psychology<br>Butler, Siobhan; Helen and Douglas House<br>Smith, Colette; Oxford Health NHS Foundation Trust<br>Lee, Alex CH; Oxford University Hospitals NHS Foundation Trust<br>Vincent, Charles A; University of Oxford, Experimental Psychology
Keywords:	Gastroenterology, Health services research, Nursing

SCHOLARONE™
Manuscripts

I, the Submitting Author has the right to grant and does grant on behalf of all authors of the Work (as defined in the below author licence), an exclusive licence and/or a non-exclusive licence for contributions from authors who are: i) UK Crown employees; ii) where BMJ has agreed a CC-BY licence shall apply, and/or iii) in accordance with the terms applicable for US Federal Government officers or employees acting as part of their official duties; on a worldwide, perpetual, irrevocable, royalty-free basis to BMJ Publishing Group Ltd ("BMJ") its licensees and where the relevant Journal is co-owned by BMJ to the co-owners of the Journal, to publish the Work in this journal and any other BMJ products and to exploit all rights, as set out in our licence.

The Submitting Author accepts and understands that any supply made under these terms is made by BMJ to the Submitting Author unless you are acting as an employee on behalf of your employer or a postgraduate student of an affiliated institution which is paying any applicable article publishing charge ("APC") for Open Access articles. Where the Submitting Author wishes to make the Work available on an Open Access basis (and intends to pay the relevant APC), the terms of reuse of such Open Access shall be governed by a Creative Commons licence – details of these licences and which Creative Commons licence will apply to this Work are set out in our licence referred to above.

Other than as permitted in any relevant BMJ Author's Self Archiving Policies, I confirm this Work has not been accepted for publication elsewhere, is not being considered for publication elsewhere and does not duplicate material already published. I confirm all authors consent to publication of this Work and authorise the granting of this licence.

Confidential: For Review Only

Training and support for caring for a child's gastrostomy:

A parental survey

Bethan Page[1], Siobhan Butler[2], Colette Smith[3] Alex CH Lee[4], Charles A Vincent[1]

1 Department of Experimental Psychology, New Radcliffe House, Radcliffe Observatory Quarter, Woodstock Road, Oxford, OX2 6AE, UK

2 Helen & Douglas House, Oxford, UK

3 Oxford Health NHS Foundation Trust, UK

4 Oxford University Hospitals NHS Foundation Trust, UK

Correspondence to

Bethan Page, Department of Experimental Psychology, University of Oxford, New Radcliffe House, Radcliffe Observatory Quarter, Oxford, UK, OX2 6GG, Bethan.page@psy.ox.ac.uk

WORD COUNT: 2973

Confidential: For Review Only

ABSTRACT

Objective

The aim of this study was to explore parents' experiences of training and ongoing support for caring for their child's gastrostomy, and to get their views on how this could be improved.

Methods

A mixed-methods online survey with 146 families who care for a child with a gastrostomy. Parents rated their own experience of training and support and made recommendations for how training and support could be improved for future parents.

Results

Two in five parents rated their confidence caring for their child's gastrostomy as very low in the first few weeks after surgery. Many parents felt that demonstrations in hospital were too brief. The quality of support parents received in the community varied. Parents benefitted from ongoing learning and support from other parents. Videos and simulation practice were rated as useful, in addition to face-to-face supervised practice with a clinician. Parents liked how real life the example video shown was, and rated nearly all suggested video topics as "very helpful", especially troubleshooting topics.

Conclusions

The training parents receive to care for their child's gastrostomy varies considerably. Many parents felt that their training was too basic or brief, and did not prepare them for managing problems. Improved training and support, e.g. through instructional videos, could make a substantial difference to parents' experiences, reduce the risk of harm to children and reduce demand on NHS services. Training needs to begin prior to the child's surgery and be regarded as an essential component of the care package.

Confidential: For Review Only

What is already known on this topic

- It is now commonplace for parents to carry out complex medical procedures for children at home.
- Some studies have reported safety concerns around the practices of parents caring for children with gastrostomies

What this study adds

- Training for parents is variable, and sometimes poor, leaving many parents feeling anxious and under-confident.
- Parents valued supervised practice with healthcare professionals, videos featuring parents and clinicians and more hands on practice, including using models/dolls.
- Training in a short hospital admission is often insufficient to prepare parents.
- Training should begin prior to the hospital admission for surgery and be regarded as an essential component of the care package.

Confidential: For Review Only

Confidential: For Review Only

INTRODUCTION

Many medical procedures, which were once carried out only by healthcare professionals in hospital wards, are now performed by families at home.[1-3] There are increasing numbers of children with complex medical needs.[4,5] Their parents perform a range of medical procedures for them at home, including feeding tube care, home oxygen, tracheostomy care and bowel washouts.[1,4,6] There are numerous benefits for families and the healthcare system when children can be cared for at home, rather than in healthcare settings,[7,8] but there are also substantial risks.[2] An analysis of incident reports on enteral tube feeding identified training and support for parents as a weakness in the system.[9] Without consistently high-quality training and support for parents, children are at risk of harm, and parents feel under-confident, anxious and alone.[6,10]

Caring for a child with a gastrostomy

A gastrostomy is a surgically placed device where feeds are delivered directly into the stomach. Gastrostomies are common in children with severe chronic illnesses and neurodisability who have difficulty swallowing or cannot get adequate nutrition through eating and drinking.[11,12] Parents have to learn to administer feeds, water and medications via the tube, to clean and care for the stoma site, and to manage complications such as leakage and blocked tubes. Parents may also learn to change a gastrostomy button. Parents normally receive some bedside teaching in hospital (during a hospital stay of around two days), with ongoing support from community nurses, but this may not be sufficient to ensure safe care at home. There are some safety concerns reported in the literature around the practices of parents caring for children with gastrostomies.[9,13,14] In a recent study on same-day discharge for gastrostomy surgery, the most common reason for a delayed discharge was parents not feeling sufficiently confident caring for the gastrostomy.[15] Improving training and support for families could reduce callouts to over-stretched community teams, as well as preventing avoidable Emergency Department visits and admissions, e.g. through appropriate timely management of a dislodged button.

Many parents caring for children with complex medical needs develop substantial expertise over time.[6,9,10] However training for parents can be informal and highly variable [6,10,11]. This contrasts with training for healthcare professionals who receive years of university training, practice-based training and ongoing continuing professional development (CPD). Healthcare professionals typically also have on-site backup and support, whereas families are often home alone whilst carrying out medical tasks.[16] Good quality training for parents is key for optimising outcomes for children.[17] The aim of this study is to explore parents' experiences of training and ongoing support for caring for their child's gastrostomy, and to find out their views on how training and support could

be improved. This paper is a part of a longer-term project to develop a package of training and support for families.

METHODS

Survey aims

The first aim was to understand parents' experiences of training and support for caring for their child's gastrostomy. The second aim was to understand parents' recommendations for improving training and support.

Survey development

The survey was developed in stages. In the initial exploratory stage, we reviewed the literature on gastrostomy training and consulted parent representatives, nurses and paediatricians from the community and hospital. The content of the survey was informed by findings from a preliminary qualitative survey with 50 families who performed a range of medical procedures, which highlighted parents' feelings of being scared and unprepared, the variability in experiences of training and the emotional demands on parents. The survey instrument was drafted, piloted and revised in consultation with parent representatives, children's nurses and paediatricians from the community, a paediatric gastrointestinal surgeon and a specialist surgical nurse. The team were asked to comment on the suitability of the questions, readability and length. Recruitment strategies were developed based on advice from our parent representatives, clinicians and charities.

Survey design and content

The survey was a fully mixed-methods survey with the qualitative data intended to complement, illustrate and expand the quantitative data.[18] The survey tool used was Qualtrics. The survey is available in Supplementary File 1.

Participants

Participants were recruited through UK charities and local charities and through our parent-representatives who posted on closed Facebook groups which serve as support groups for families. Some parents also choose to share the survey with friends/family (further information is given in Supplementary File 2). The inclusion criteria was any parent or family carer who provides gastrostomy care to a child or young person at home under 25 years. By family carer, we included any unpaid carer (parent, relative, friend) who actively participates in caring for a child with a gastrostomy. To take part family carers needed to be at least 18 years old. Participants received a £10 voucher for taking part. All participants gave informed consent before taking part and consented

Confidential: For Review Only

for their data to be used in publications (The Medical Sciences Inter-Divisional Research Ethics Committee, Oxford University, R56623/RE004). The data was collected between July and September 2020. We aimed to recruit at least 100 families to capture a broad range of experiences, including families new to gastrostomy care and some more experienced.

Analysis

Descriptive statistics were computed for all close-ended questions, using SPSS Statistics 25. Participants who did not complete the full survey were excluded. The open-ended questions were coded in NVivo 12 using inductive content analysis, to group responses based on surface level of meaning.[19] Answers were coded line by line, and grouped into categories emerging from the data. These were summarised in the text and illustrated with quotes from participants.

Patient and Public Involvement

Two parent representatives were involved in the design, conduct and dissemination of the research. The two parents attended the research meetings from conception of the project, alongside a team of multidisciplinary healthcare professionals. The recruitment strategy was devised through consultation with our parent representatives. The parents completed the draft survey, which was then revised based on their feedback and feedback from clinicians. The recommendations from the survey were developed through meetings with parents and the healthcare professionals supporting the research.

RESULTS

Participants

One-hundred-and-forty-six participants completed the full survey (defined as viewing all pages of the survey and completing all the quantitative questions at a minimum). 195 participants consented to take part and 250 people viewed the first page. The majority of the 146 participants were mothers (91%), there was a range of ages of the children and time since initial gastrostomy surgery. The most common types of devices were gastrostomy buttons and percutaneous endoscopic gastrostomy (PEG) tubes. Table 1 gives more detail about the families.

Table 1 About the families who completed the full survey

	N (%)
**Relation to child**	
Mother	133 (91%)
Father	8 (6%)
Other family member	5 (3%)

Confidential: For Review Only

**Age of children (years)**	
0-4	50 (34%)
5-9	38 (26%)
10-14	39 (27%)
15-19	18 (12%)
20-25	1 (1%)
**Time since initial gastrostomy surgery (years)**	
<1 year	15 (10%)
1-2	41 (28%)
3-4	27 (18%)
5+ years	63 (43%)
**Type of gastrostomy device child has or previously had**	
PEG tube	73 (50%)
Gastrostomy button (MINI or Mic-Key)	115 (79%)
Another device (e.g. gastro-jejunostomy (GJ) tube)	23 (16%)

Confidential: For Review Only

Parents' experiences of initial training

Parents' experiences of training were hugely variable (see Box 1). Most parents described receiving some training in hospital but the nature and extent varied considerably. Descriptions of training as "*brief*" or "*basic*" were common. Some parents felt unprepared and anxious: *"It was scary because we were worried about it getting caught/pulled and hurting or causing damage to our little boy. We didn't get much practice before being left to do it on our own so you are triple checking yourself and worrying did I do it right."* Some parents described receiving further training at home by community nurses or representatives from a feeding company. Parents described learning through other parents, often through Facebook groups: *"Also had amazing advice from other mums whose children have tubes who I found via Facebook groups - game changing stuff."*

Box 1 Example quotes illustrating the variability of training

Training sometimes brief & basic:

- *"A brief 5 mins of basics, a leaflet and home. A few weeks later a Nutricia nurse came to the house to show us the pump."*
- *"We were admitted for the surgery and spent two further days in the ward after, received very basic training on the use of the peg and left feeling absolutely terrified about using it!"*
- *"Need much more training than 10 minutes in consultant's office!"*

Primarily self-taught with support from other parents:

- *"The official training - nothing good. Absolutely disastrous, vague, unsupportive. The unofficial training and the info I sought out for myself - clear, helpful videos from other parents, useful approach of gastrostomy nurse. Properly child-centric, helpful stuff."*

More community support needed:

- *"The community nursing team rely too much on the surgical nurse to do the training and then they just catch up with a chat following any training given at the bedside. This training is not detailed/long enough for those dealing with such a complex medical needs child."*

Thorough training and support received:

- *"Our daughter was in hospital long terms due to a range of factors. We were able to observe nurses undertaking feeds, using pump, giving medication. We were also given an information pack and work book to go through, and we were observed by nurses until confident and competent to do ourselves. We were given support in community… with regards to changing button, taking care of button, and annual refresher also. Received updates with regards to how much water to inflate balloon with, frequency of Ph testing etc."*

The majority of parents reported receiving some training from their hospital team (n=115, 79%). A slightly smaller proportion received training from a Community Children's Nurse (CCN) (n=105, 72%). 35 participants (24%) mentioned training from another provider, most commonly a feeding company

(e.g. Nutricia). 82 (56%) families had received training from both the hospital and CCN team, and 8 (5%) reported not receiving training from either the hospital team or a CCN team.

Table 2 shows the types of training parents received. The most common types of training were verbal information, demonstrations and supervised practice from a healthcare professional. Very few participants received any simulation practice (hands on practice with a doll/equipment), or watched instructional videos. A few of the parents that did receive simulation practice commented on the usefulness of this: *"Really useful being able to have a little play with a tube and practice using the clip etc. before having to do it for real."*

Table 2 Types of teaching/training received by parents

**Types of teaching/training received**	**N (%)**
Given information verbally by a healthcare professional	129 (88%)
Demonstrations by a healthcare professional	125 (86%)
Practised on my child supervised by a healthcare professional	113 (77%)
Given a written booklet	85 (58%)
Simulation practice (practiced with a doll or some equipment)	19 (13%)
Directed to a website for information	13 (9%)
Demonstrations by another family member	7 (5%)
Other	5 (3%)
Videos	3 (2%)

Parents were asked how confident they felt caring for their child's gastrostomy in the first few weeks after surgery: 24 (16%) were not at all confident, 32 (22%) were slightly confident, 30 (21%) were moderately confident, 42 (29%) were mostly confident and 18 (12%) of parents said they were fully confident. At the time of the survey a majority (n=117, 80%) said they felt fully confident caring for their child's gastrostomy (n=117, 80%). The most common concern parents had were around hurting their child, caring for site after surgery, knowing "*what was normal*" in relation to the stoma site healing, worries about the tube coming out and problems such as blocked tubes or granulation: *"The first time I experienced this [granulomas] I thought my sons intestines were coming out! Nobody had ever mentioned it to me nor had I ever seen anything like it!"*

It is difficult to say from the data whether the training parents receive has improved over time, however we did not find any evidence that parents' retrospective confidence ratings from the first week at home had changed over time: 40% of parents with less than a years' experience rated themselves as 'not at all confident' or 'slightly confident' in the first week at home, compared to 38%

Confidential: For Review Only

of parents with more than 5 years' experience. There was no statistically significant association between time since gastrostomy surgery and parents' ratings of confidence in the first week at home; $\chi^2(12, N=147) = 12.06$, $p = .44$. This suggests that parents' confidence in the first week at home has not improved over time.

However as expected, there was evidence that parents' ratings of their current confidence (at the time of the survey) did improve with more years' experience: 46% of parents with less than a years' experience rated themselves as fully confident caring for their child's gastrostomy, compared to 89% of parents with more than 5 years' experience. A chi-squared test revealed a significant association between current confidence ratings and number of years since gastrostomy surgery; $\chi^2(9, N=147) = 17.54$, $p = .04$.

Ongoing support and training

Parents were asked which sources were most helpful for ongoing support (see Table 3). Contacting your community nurses was rated as "very helpful" by a majority, with less than a quarter rating hospital teams as a very helpful source of support. Notably conversations with other parents was rated as "very helpful" by 56%, with a further 32% ratings support from parents as "quite helpful". Parents in the survey offered advice for other parents, including tips for managing their child's distress when changing a gastrostomy button.

Table 3 Sources of ongoing support: how helpful are they?

	Very helpful	Quite helpful	Not very helpful	Not applicable
Contacting your community nurse	90 (62%)	28 (19%)	13 (9%)	15 (10%)
Contacting your hospital team	34 (23%)	37 (25%)	38 (26%)	37 (25%)
Facebook groups	54 (37%)	67 (46%)	11 (8%)	14 (10%)
Conversations with other parents of children with similar needs	82 (56%)	47 (32%)	3 (2%)	14 (10%)
Written information booklets provided by a healthcare professional	28 (19%)	68 (47%)	32 (22%)	18 (12%)
NHS websites	15 (10%)	55 (38%)	52 (36%)	24 (16%)
Charities	25 (17%)	53 (36%)	19 (13%)	49 (34%)
Videos	29 (20%)	50 (34%)	17 (12%)	50 (34%)

Even experienced parent carers considered that some further training might be helpful, with 14 (10%) saying definitely yes to further training or support, 29 (20%) probably yes, 81 (55%) probably

Confidential: For Review Only

not and 21 (14%) definitely not. The most common request was help with managing problems. A number of parents wanted refresher training, or updates on the latest guidance: "*making sure bad habits have not crept in and that we are up-to-date with any changes in how things are done.*"

Parents' recommendations for improving training

Participants were asked which formats of training might be useful to other parents facing the challenge of caring for a child with a gastrostomy (see Table 4). Around three-quarters rated demonstrations and practice on my child supervised by a healthcare professional as extremely useful. Around two-thirds of parents felt that videos and simulation practice would be extremely or very useful. Parents felt that these additional forms of training would be a good addition to face-to-face training with a professional, but not a substitute: *"I think that the videos are good resources that parents can go back to however I think that face to face training is really important initially."*

Table 4 Which types of training might be helpful to other parents

	Extremely useful	Very useful	Moderately useful	Slightly useful	Not at all useful
Demonstrations by a healthcare professional	105 (72%)	33 (23%)	6 (4%)	2 (1%)	0 (0%)
Practised on my child supervised by a healthcare professional	110 (75%)	29 (20%)	5 (3%)	1 (1%)	1 (1%)
Written booklets	32 (22%)	39 (27%)	51 (35%)	19 (13%)	5 (3%)
Videos	40 (27%)	58 (40%)	29 (20%)	14 (10%)	5 (3%)
Simulation training (practicing with a doll or some equipment)	47 (32%)	45 (31%)	36 (25%)	10 (7%)	7 (5%)
Online training	18 (12%)	33 (23%)	47 (32%)	30 (21%)	17 (12%)
Group training with other parents	20 (14%)	34 (23%)	39 (27%)	31 (21%)	21 (14%)
Training by an experienced parents	15 (10%)	16 (11%)	32 (22%)	51 (35%)	32 (22%)

Parents had many suggestions for improving training. A common suggestion was more training and information about common problems including what to do if the button comes out, and requests for specific types of training, such as more hands on training. Some parents commented on the timing of training as they struggled to take information on board while in hospital: *"I think we should have had*

https://mc.manuscriptcentral.com/bmjpo

Confidential: For Review Only

more [training] before his surgery and not while he was in theatre as we were so anxious I'm not sure how much we took in or how valuable doing it then was." A few parents said they would like to have been put in touch with other families: *"Opportunity to do group talks or training would be good. Nice way to meet other families and build a support network."*

Parents' recommendations for video development

Parents were shown a sample video showing a parent administering a bolus of water to their child. Parents liked how "real life" the video was: the child was wriggling during the procedure, it was done at home, it was relaxed and the mother was talking to the child throughout. One parent commented that: "*I've hated the teaching videos previously as they seem rather clinical but this was fantastic*." When asked where training videos should be filmed, 54 (37%) said at home, 4 (3%) said in hospital, and 88 (60%) said a mixture of both locations. 116 (80%) of participants wanted both healthcare professionals and parents to feature, 12 (18%) wanted just healthcare professionals and 8 (12%) wanted just parents. Parents rated different topics for videos: the vast majority were rated as 'very helpful' (see Supplementary file 3).

DISCUSSION

The training parents receive to care for their child's gastrostomy varies considerably, and is sometimes of poor quality. Parents are often anxious in the first few weeks of caring for their child at home and concerned about doing something wrong or hurting their child. Demonstrations in hospital were frequently described as too brief, and ongoing support in the community was highly variable. Parents valued support from other parents. Videos and simulation practice were rated as useful preparation, in addition to face-to-face supervised practice with a healthcare professional. Parents wanted videos to feature parents and health professionals and for at least some of the videos to be filmed at home.

Implications for the design of services

Shorter hospital stays are increasingly advocated for gastrostomy surgery, including same day discharges,[15] meaning that there is little time to train parents during admission. Recommendations from parents suggest that preparation and training needs to begin before admission, with time during or after admission for parents to practice on their child supervised by designated professionals. At present, many parents do not feel confident caring for their child when they first go home after surgery. Parents ought to receive more preparation for caring for their child's gastrostomy from either hospital or community nurses before surgery takes place: this has the potential to improve parents' confidence ratings in the first few weeks at home after surgery. Instructional videos and written materials should be available to all families awaiting gastrostomy

Confidential: For Review Only

surgery, to be revisited as needed. To enable more opportunity for hands-on practice, parents could practice with dolls and equipment at home: there is substantial evidence on the benefits of repeated hand-on-practice in the medical education literature.[20,21] There is potential to make more use of online training and support, including group video-calls for families. Training resources should be co-designed with families and address their emotional needs as well as the technical aspects of care. Box 2 gives an overview of practical suggestions for improving training based on discussions on the survey data with our multidisciplinary group of healthcare professionals and parents. We are currently creating a library of videos co-produced with families, paediatricians and nurses from the hospital and community and piloting hands-on practice with equipment and 3D-printed models.

Box 2: Practical suggestions for improving training based on parents' descriptions of what they liked about their current training and their recommendations for improvement.

Before surgery

- Parents watch videos teaching them about routine care of a gastrostomy, how to manage common problems, and advice and tips from more experienced families. The videos should to be accessible to families who do not speak fluent English, and feature families from different cultural backgrounds
- Parents receive a home visit from community nurse for face-to-face teaching with models and to ask questions
- Parents repeatedly practice with a doll/equipment to become familiar with the basics before their child's surgery, e.g. connecting extension tube
- Parents invited to a group call with other families awaiting surgery to ask questions to surgical team and meet other parents
- Parents directed to Facebook groups and other peer support options

During hospital admission

- Supervised practice doing procedures on child after surgery with support of designated nurse

After hospital admission

- Community nurses provide further support and teaching through home visits and video-calls
- Parents can revisit videos and written materials as needed including videos on managing common problems such as granulation
- Parents supported by community nurses to learn to change a button, through repeated practicing with models in addition to supervised changes on their child

Strengths and limitations

The sample was recruited predominantly through charities and Facebook groups. This enabled us to recruit a large sample of families from across the country, to ensure experience of a wide range of clinical services. The survey included families new to gastrostomy care and very experienced families. It is possible there are some issues of recall in parents who received their training a long time ago (43% of parents had more than 5 years' experience of caring for their child's gastrostomy). It is difficult to know how representative the samples are of all families: we may have recruited families who are more engaged in their child's care or families who felt unprepared and sought help through Facebook groups and charities. We did not collect demographic data on the families so cannot tell the socio-economic, health literacy or ethnicity of families. We also did not collect data on the children's diagnoses or the level of medical complexity, although we purposely advertised through charities that support children with a range of different diagnoses and levels of complexity. Families were very engaged and many offered to help produce videos. Future research is needed to understand healthcare professionals' views on training families and ideas for improvement, to compare against the findings from the families in this survey.

CONCLUSIONS

Training for parents to care for their child's gastrostomy is highly variable and sometimes of poor quality. Parents value face-to-face training with a healthcare professional, videos which show "real-life" featuring families and clinicians and hands-on practice including with dolls/equipment. There is much that can be done to support and train families which need not involve substantial staff time and resources, and can improve the lives of families and reduce the risk of problems and complications which cause harm to children and increase demand on NHS resources. There is huge value in involving families in designing improvements to services, and treating them as equal partners in children's care.

Confidential: For Review Only

REFERENCES

1. Ten Haken I, Ben Allouch S, Van Harten WH. The use of advanced medical technologies at home: A systematic review of the literature. *BMC Public Health* 2018;**18**(1):284.

2. Institute for Healthcare Improvement. No Place Like Home: Advancing the Safety of Care in the Home 2018. Available from: http://www.ihi.org/resources/Pages/Publications/No-Place-Like-Home-Advancing-Safety-of-Care-in-the-Home.aspx

3. Reinhard S, Levine C, Samis S. Home alone: family caregivers providing complex care. *Gerontologist* 2017;**56**(Suppl_3):636–636.

4. Cohen E, Berry JG, Sanders L, Schor EL, Wise PH. Status Complexicus? The Emergence of Pediatric Complex Care. *Pediatrics* 2018;**141**(Suppl 3):S202–S211.

5. Fraser L, Gibson-Smith D, Jarvis S, Norman P, Parslow R. "Make Every Child Count" Estimating current and future prevalence of children and young people with life-limiting conditions in the United Kingdom. 2020. Available from: https://www.togetherforshortlives.org.uk/resource/make-every-child-count/

6. Page BF, Hinton L, Harrop E, Vincent C. The challenges of caring for children who require complex medical care at home: 'The go between for everyone is the parent and as the parent that's an awful lot of responsibility.' *Heal Expect* 2020;**23**(5):1144-1154.

7. Glasson EJ, Forbes D, Ravikumara M, Nagarajan L, Wilson A, Jacoby P, et al. Gastrostomy and quality of life in children with intellectual disability: A qualitative study. *Arch Dis Child* 2020, **105**(10):969-974.

8. Mitchell TK. The impact of medical technology upon the home and feelings of at-homeness for children and young people with complex health care needs and their family members. PhD Thesis Tracy Karen Mitchell, Edge Hill University. 2020. Available at: https://research.edgehill.ac.uk/en/studentTheses/the-impact-of-medical-technology-upon-the-home-and-feelings-of-at

9. Page B, Nawaz R, Haden S, Vincent C, Lee ACH. Paediatric enteral feeding at home: An analysis of patient safety incidents. *Arch Dis Child* 2019;**104**:1174-1180.

10. Spiers G, Beresford B. "It goes against the grain": A qualitative study of the experiences of parents' administering distressing health-care procedures for their child at home. *Heal Expect* 2017; **20**(5):920-928.

Confidential: For Review Only

11. Burdall OC, Howarth LJ, Sharrard A, Lee ACH. Paediatric enteral tube feeding. *Paediatrics and Child Health* 2017; **27**(8):P371-377,2017.

12. Puntis JWL. Benefits and management of gastrostomy. *Paediatrics and Child Health* 2009;**19**(9):415–24.

13. Evans S, Shelton F, Holden C, Daly A, Hopkins V, MacDonald A. Monitoring of home safety issues in children on enteral feeds with inherited metabolic disorders. *Arch Dis Child* 2010; **95**(9): 668–672.

14. Evans S, Preston F, Daly A, Ashmore C, MacDonald A, Holden C. Home enteral tube feeding in children with inherited metabolic disorders: a review of long-term carer knowledge and technique. *J Hum Nutr Diet* 2012;**25**(6):520-525.

15. Dekonenko C, Svetanoff WJ, Osuchukwu OO, Pierce AL, Orrick BA, Sayers KL, et al. Same-day discharge for pediatric laparoscopic gastrostomy. *J Pediatr Surg* 2020; **56**(1):26-29.

16. Reinhard, S., Levine, C., & Samis, S. (2012). HOME ALONE: Family Caregivers Providing Complex Chronic Care. In *AARP Public Policy Institute*.

17. Berman L, Hronek C, Raval M V, Browne ML, Snyder CL, Heiss KF, et al. Pediatric gastrostomy tube placement: Lessons learned from high-performing institutions through structured Interviews. *Pediatr Qual Saf* 2017;**2**(2):e016.

18. Terry G, Braun V. Short but Often Sweet. In: Collecting Qualitative Data. Cambridge University Press; 2017.

19. Elo S, Kyngäs H. The qualitative content analysis process. *J Adv Nurs* 2008;**62**(1):107–15.

20. Motola I, Devine LA, Chung HS, Sullivan JE, Issenberg SB. Simulation in healthcare education: A best evidence practical guide. AMEE Guide No. 82. *Med Teach* 2013;**35**(10):142–59.

21. McGaghie WC. Does Simulation-based Medical Education with Deliberate Practice Yield Better Results than Traditional Clinical Education? A Meta-Analytic Comparative Review of the Evidence. *Acad Med* 2012;**86**(6):706–11.

Confidential: For Review Only

Acknowledgements

We would like to thank all the parents who completed the survey. We are grateful for the expertise of our multidisciplinary stakeholder group, which includes parents, paediatricians, children's nurses and specialist surgical nurses. We thank the team for their ongoing work developing training for parents. This work was supported a grant from Health Foundation's Q Exchange Programme. BP is supported by the Economic and Social Research Council [grant number ES/J500112/1]. CV is supported by the Health Foundation.

Contributorship statement

BP and CV conceived and designed the study. The survey was designed and revised by BP with support from SB, CS, AL & CV. BP analysed the data. BP drafted the manuscript with support from AL, SB, CS and CV. All authors provided critical feedback on the manuscript and analysis. All authors approved the final manuscript.

Competing interests

None declared.

Data sharing statement

Data may be obtained upon reasonably request by contacting the corresponding author. The survey tool used is available in the supplementary information.

Confidential: For Review Only

Supplementary File 1: Survey Tool

Overview of survey

Gastrostomy care: an online survey for parents on training and support needs

- This survey is for family carers (e.g. mums, dads, grandparents) who care for a child or young person who has a gastrostomy (e.g. PEG or button).
- This survey is part of a project to develop resources to better prepare and support families who are new to gastrostomy care. The project is a collaboration between researchers, parents and healthcare professionals.
- The findings will be shared widely with parents, charities, healthcare providers and researchers.

What do I have to do to take part?

- This study is an online survey and will take 15-20 minutes to complete.
- You will be asked about your experiences of learning to care for your child's gastrostomy, your experiences of ongoing support and what videos and resources might be helpful.
- To take part, the child you care for needs to be under 18 years and you need to be at least 18 years old.
- Please read the participant information sheet on the next page if you are interested in taking part.

Thank you for your time.

Page Break

Confidential: For Review Only

Participant information

We [the University of Oxford in collaboration with clinicians and parent representatives] are investigating the experiences and training needs of family carers in the UK who provide specialist healthcare to children at home, in order to develop resources for training and supporting other families. We are currently creating videos and resources for parents new to gastrostomy care and are seeking your feedback on how we should do this.

We appreciate your interest in participating in this online survey. You have been invited to participate as you are a parent or a family carer who provides gastrostomy care to a child or young person under 18 at home. By family carer, we include any unpaid carer (parent, relative, friend, foster carer) who actively participates in caring for a child or young person with a gastrostomy. Tasks you might do include giving feeds, medications, caring for the stoma site and/or changing a gastrostomy button. To take part you, the family carer, need to be at least 18 years old.

Please read through this information before agreeing to participate by ticking the 'yes' box below.

You will be asked questions on:

i) you and your child (e.g. child's age, your relationship to the child)

ii) your experiences of learning and being trained to care for your child's gastrostomy

iii) your experiences of ongoing support with caring for your child's gastrostomy

iv) what training and support would help other parents

v) what videos would be helpful

The survey should take 15-20 minutes to complete. No background knowledge is required.

Confidential: For Review Only

How will your data be used?

The survey will be used to inform the development of videos and other resources to support and prepare families new to gastrostomy care. Some of the videos will also be helpful to more experienced parents and for training staff.

The study results will be written up in a DPhil (PhD) thesis and potentially also in an academic publication. Key findings will be shared widely with participants, family carers, charities, health care professionals and researchers.

Your answers will be completely anonymous, and we will use all reasonable endeavours to keep them confidential. You will be given the option at the end of the survey to provide us with your email address if you would like to receive a summary of the research findings or if you would be interested in helping us rate and evaluate the videos we are creating. Email addresses and telephone numbers will be stored on a password protected file on a secure university server. This is part of a longer term project to develop new methods for training and supporting family carers.

Please note that your participation is voluntary. You may withdraw at any point during the survey for any reason, before submitting your answers, by closing the browser. If you need to leave the survey before you have time to fully complete it, you can return to finish your partially completed survey using the same device (tablet, computer, mobile) within one week of starting it. After one week, your data will be saved and you will be unable to continue the survey.

Your data will be stored in a password-protected file on secure university servers and may be used in academic publications. Your IP address will not be stored. All questions are optional.

Who will have access to your data?

The University of Oxford is the data controller for the purposes of the Data Protection Act 1998. Your information may be shared with members of the research team. This questionnaire is for a DPhil [PhD] project. The principal researcher is Bethan Page, who is attached to the Department of Experimental Psychology at the University of Oxford. This project is being completed under the supervision of Professor Charles Vincent, Professor Nick Yeung and Dr Emily Harrop (clinical lead for the project).

Confidential: For Review Only

This project has been reviewed by, and received ethics clearance through, the University of Oxford Central University Research Ethics Committee [reference number R56623/RE001].

Participant benefits and risks

If you complete the full survey, we will email you a £10 Marks and Spencer (M&S) voucher as a thank you for taking part. You will be asked to leave your email address at the end of the survey so we can send you the voucher. Your email address will be deleted from our records after we have sent you the voucher (unless you have consented to be contacted in the future with a summary of the findings, or to help with future studies).

There will be no other direct benefits to you in taking part in this study. However, you may find it useful to share your experiences and views on improving training and support. Your involvement will improve our understanding of current training provision and support needs of family carers. Key findings will be used to develop resources to better support and prepare family carers, and will be shared widely with families, healthcare providers, charities and researchers.

There are no direct risks involved in taking part in this study. However it is possible that you may find some of the topics distressing or difficult. You don't have to answer all the questions and you can exit the survey at any point or withhold any information that you do not wish to share. A list of website and charities offering further support will be provided at the end of the survey.

What if there is a problem?

If you have a concern about any aspect of this project, please contact the researcher Bethan Page (email: bethan.page@psy.ox.ac.uk) or their supervisor Professor Charles Vincent (charles.vincent@psy.ox.ac.uk) who will do their best to answer your query. If your problem is a clinical query, we will contact the clinical lead for the project, Dr Emily Harrop (Consultant in Paediatric Care) for guidance.

The researcher should acknowledge your concern within 10 working days and give you an indication of how they intend to deal with it. If you remain unhappy or wish to make a formal

Confidential: For Review Only

complaint, please contact the relevant Chair of the Research Ethics Committee at the University of Oxford:

Chair, Medical Sciences Inter-Divisional Research Ethics Committee;

Email: ethics@medsci.ox.ac.uk;

Address: Research Services, University of Oxford, Wellington Square, Oxford OX1 2JD

The Chair will seek to resolve the matter in a reasonably expeditious manner.

If you have any questions about the survey please email bethan.page@psy.ox.ac.uk.

Please note that you may only participate in this survey if you are 18 years of age or over.

○ I am 18 years or over

If you have read the information above and agree to participate with the understanding that the data (including any personal data) you submit will be processed accordingly, please check the relevant box below to get started.

○ Yes I agree to take part

Confidential: For Review Only

About you and your child

Section 1 of 5:
In this first section we would like to ask some questions about you and the child or young person you provide care for.

Q1.1 What is your relationship to the child that you care for?

◯ Mother (1)

◯ Father (2)

◯ Other [please specify] (4) ____

Q1.2 How old is your child?

____

Q1.3 How long ago did your child first have gastrostomy surgery?

◯ Less than a year (1)

◯ 1 - 2 years (2)

◯ 3 - 4 years (3)

◯ 5 + years (4)

Confidential: For Review Only

Q1.4 Which types of gastrostomy tube has your child had? [select all that apply]

☐ Gastrostomy button (e.g. MIC-KEY button, MINI button) (1)

☐ Percutaneous Endoscopic Gastrostomy (PEG) (e.g. Freka, Corflo) (2)

☐ Other [please specify] (3) ____

Experiences of learning and being trained

Section 2 of 5:
In this section we would like to understand what training and information you received to care for your child's gastrostomy and what you found helpful.

Parents have to learn to give feeds and medications, clean and care for the stoma site and learn what to do if there is a problem, such as if the PEG tube blocks or the gastrostomy button comes out. There are also other tasks you may have learnt to do such as changing a gastrostomy button.

Q2.1 Tell us about what training and information you received when your child first had gastrostomy surgery.

____

____

____

____

____

Q2.2 Tell us about any training or information you have received since the initial surgery.

____

____

Confidential: For Review Only

____

____

____

Q2.3 Who have you received training or information from? [select all that apply]

☐ Hospital team (1)

☐ Community Children's Nurse (CCN) (2)

☐ Other [please specify] (3) ____

Q2.4 What type of training/teaching have you received? [select all that apply]

☐ Given information verbally by a healthcare professional (23)

☐ Demonstrations by a healthcare professional (24)

☐ Practised on my child supervised by a healthcare professional (32)

☐ Demonstrations by another parent or family member (39)

☐ Given a written booklet (33)

☐ Directed to a website for information (34)

☐ Simulation practice (e.g. practised with a doll or some equipment) (35)

☐ Videos (38)

☐ Other [Please state] (37) ____

Confidential: For Review Only

Q2.5 What was good about the training you received to care for your child's gastrostomy?

____

____

____

____

____

Q2.6 What could have been improved about your training or was there anything missed?

____

____

____

____

____

Q2.8 How confident did you feel to care for your child's gastrostomy **in the first week** at home after their surgery?

- ○ Not at all confident (1)
- ○ Slightly confident (2)
- ○ Moderately confident (3)
- ○ Mostly confident (4)
- ○ Fully confident (5)

Confidential: For Review Only

Q2.9 What were you not confident with?

____

____

____

____

____

Experiences of ongoing support

Section 3 of 5:
In this section we would like to understand how confident you feel now and what ongoing support you have found helpful or might want.

Q3.1 How confident do you feel **now** caring for your child's gastrostomy?

- ○ Not at all confident (1)
- ○ Slightly confident (2)
- ○ Moderately confident (3)
- ○ Mostly confident (4)
- ○ Fully confident (5)

Confidential: For Review Only

Q3.2 Would it be helpful to receive any further training or support?

- Definitely yes (1)
- Probably yes (2)
- Probably not (3)
- Definitely not (4)

Q3.3 If yes, what would you like further training or support with?

Confidential: For Review Only

Q3.4 How helpful do you find the following sources for ongoing information and support? (Please select all that apply)

	Very helpful	Quite helpful	Not very helpful	Not applicable
Contacting your community nurse	○	○	○	○
Contacting your hospital team (e.g. specialist nurse)	○	○	○	○
Facebook groups	○	○	○	○
Conversations with other parents of children with similar needs	○	○	○	○
Written information/booklets provided by a healthcare professional	○	○	○	○
NHS websites	○	○	○	○
Charities	○	○	○	○
Videos	○	○	○	○
Other [please state]	○	○	○	○

Confidential: For Review Only

Section 4 of 5: *We are working to develop training and resources for parents new to gastrostomy care.* In this section we would like to get your views on what training and support is most helpful to parents generally.

Q4.1 Which formats for training do you think are most useful?

	Extremely useful	Very useful	Moderately useful	Slightly useful	Not at all useful
Demonstrations by healthcare professionals	○	○	○	○	○
Practice supervised by a healthcare professional	○	○	○	○	○
Written booklets	○	○	○	○	○
Videos	○	○	○	○	○
Simulation training (e.g. hands on practice with a doll or some equipment)	○	○	○	○	○
Online training	○	○	○	○	○
Group training sessions with other parents	○	○	○	○	○
Training by an experienced parent	○	○	○	○	○

Confidential: For Review Only

Q4.2 If you use Facebook groups for ongoing support relating to your child's gastrostomy, are there any you would recommend to other parents? If yes, please name.

Q4.3 We know that some children and parents find some tasks distressing, such as changing a gastrostomy button. Do you have tips to share with parents, or know of any resources that might be helpful to other parents?

Section 5 of 5: *We are developing some training videos for parents who are new to gastrostomy care. Some of the videos may also be helpful to more experienced parents and for training staff. In this final section we would like to get your views on what these videos should look like.*

Q5.1 We have started creating some videos. Here is an example video:

Confidential: For Review Only

Q5.2 What did you like about the video?

Q5.3 What could be improved or was missing in the video?

Confidential: For Review Only

Q5.4 Which of the following videos do you think would be helpful to other parents?

	Really helpful	Quite helpful	Not very helpful	Not applicable to me
Caring for the stoma/gastrostomy site	○	○	○	○
Flushing the tube	○	○	○	○
Giving a feed	○	○	○	○
Giving medications	○	○	○	○
Venting (letting air or gas out)	○	○	○	○
Changing the water in the balloon of the gastrostomy button	○	○	○	○
How to change a gastrostomy button	○	○	○	○
What to do if the tube blocks	○	○	○	○
What to do if the tube comes out	○	○	○	○

Confidential: For Review Only

Q5.5 Some of the videos will cover the technical aspects of care. We also recognise that there are wider topics which parents might find helpful. Which of the suggestions below might be helpful?

	Really helpful	Quite helpful	Not very helpful
Recommendations for Facebook groups to join	○	○	○
Tips for managing your child's distress, e.g. when changing a button	○	○	○
Advice for new parents from more experienced parents	○	○	○

Q5.6 Do you have any other suggestions for topics or things we should consider?

____

____

____

____

____

Q5.7 Where should the videos be filmed?

○ Home

○ Hospital

○ Mixture

Confidential: For Review Only

Q5.8 Who should be delivering the teaching/information in the videos?

- ◯ Parents
- ◯ Healthcare professionals
- ◯ Mixture

Q5.9 Do you have any final comments for us on videos or other resources to support parents?

____

____

____

____

____

Thank you very much for your time today. Your thoughts and advice will be really helpful to us as we develop resources for preparing and supporting families.

Do you have any final comments for us?

____

____

____

____

____

As a thank you for your help we will be sending all participants who complete the full survey a voucher (£10 M&S e-giftcard). Please leave your email address here so we can send it to you (we will aim to email it to you within two weeks).

____

Confidential: For Review Only

Would you like to be emailed a summary of the findings from this project?

- ◯ Yes
- ◯ No

We are currently developing more videos for families new to gastrostomy care. The videos will also be used for training staff. We will need help from parents later in the year to rate/evaluate the videos and resources.

Would you be willing to do this? We will compensate you for your time.

- ◯ Yes, please contact me to help rate/evaluate more videos
- ◯ No, please don't contact me about this.

Below is a link to a list of charities and resources which you may find helpful as a source of information and support. If this survey has raised any questions or concerns about the care of your child, please contact a healthcare professional for advice: Charities and other resources

Confidential: For Review Only

Further details about the survey

For all the closed-ended/quantitative questions we used qualtrics' 'request response' option. This function rem was unanswered questions on the page before they could move on to the next page, but it did not force partici ended qualitative questions did not request a response. We included participants who completed all the quanti
The back button was enabled so participants could go back and check their answers.
We did not do any formal checks for unique site visitors. We identified one participant who had completed the email addresses submitted for the vouchers. The participants' second response was excluded.

Confidential: For Review Only

Supplementary File 2: Parents' recommendations for topics for videos

Parents were asked to rate various topics for videos on a three point scale with 1 indicating 'not very helpful' and 3 indicating 'very helpful'. The vast majority of topics were rated as 'very helpful', with the lowest mean score being 2.45. Topics related to problem solving were generally rated as slightly more helpful than routine care.

Table 1 Parents rating of which topics would be most helpful to cover in videos

	Very helpful	Quite helpful	Not very helpful	Not applicable
What to do if the tube comes out	129 (88%)	13 (9%)	1 (1%)	3 (2%)
What to do if the tube blocks	126 (86%)	17 (12%)	1 (1%)	2 (1%)
How to change a gastrostomy button	118 (81%)	18 (12%)	2 (1%)	8 (5%)
Venting (letting air or gas out)	114 (78%)	23 (16%)	1 (1%)	8 (5%)
Changing the water in the balloon of the gastrostomy button	110 (75%)	27 (18%)	1 (1%)	8 (5%)
Advice for new parents from more experienced parents	115 (79%)	27 (18%)	4 (3%)	0 (0%)
Caring for the stoma/gastrostomy site	106 (73%)	37 (25%)	1 (1%)	2 (1%)
Tips for managing child's distress (e.g. when changing button)	100 (68%)	37 (25%)	8 (5%)	1 (1%)
Giving a feed	94 (64%)	48 (33%)	3 (2%)	1 (1%)
Giving medications	93 (64%)	50 (34%)	3 (2%)	0 (0%)
Recommendations of Facebook groups to join for support	82 (56%)	52 (36%)	11 (8%)	1 (1%)
Flushing the tube	76 (52%)	57 (39%)	11 (7.6%)	2 (1%)

Confidential: For Review Only

Parents' suggestions of other topics to include

Parents had a wide range of suggestions for other topics for videos. Common technical topics included blended diet, tips for specific problems (e.g. replacing connector end of Freka peg) and different types of feeding tubes. They also had suggestions relating to wider support needs, including advice on doing daily activities, ways of explaining feeding tubes to children and siblings and reassuring other parents that children can still do normal activities: "*Showing people that they can get out and about with their child still. I know parents who return home to feed their child it would be nice to show how a gtube doesn't stop fun*."

Confidential: For Review Only

Supplementary File 3 : Additional information on recruitment

Advertising the survey

Participants were recruited through UK charities and local charities and through our parent-representatives who posted on Facebook parent support groups. Some parents also choose to share the survey with friends/family.

The advertisement information informed participants that we were looking for family carers (e.g. parents) who cared for children with gastrostomies to complete a survey on their training and support needs. The exact wording varied slightly depending between the different charities and posts on Facebook. The first page of the survey gave some brief information about the survey (see appendix).

Web/email

The survey was posted on websites and social media sites. Some charities choose to email their parent groups.

Context

We recruited through closed Facebook groups which act as support groups for families. For example 'Tube Feeding your child in the UK' which has 4105 members, and the 'Blended Diet UK' group has 4200 members. Well Child, TOFs and Together for Short Lives are all charities who support families caring for children with complex medical needs, some of whom have feeding tubes: these charities all helped with recruitment for the survey distributed the link via email or in their closed Facebook groups. It is difficult to know how representative the samples are of all families: we may have recruited families who are more engaged in their child's care or families who felt unprepared and sought help through Facebook groups and charities.

Confidential: For Review Only

BMJ Paediatrics Open

Training and support for caring for a child's gastrostomy: A survey with family carers

Journal:	*BMJ Paediatrics Open*
Manuscript ID	bmjpo-2021-001068.R2
Article Type:	Original research
Date Submitted by the Author:	09-Jul-2021
Complete List of Authors:	Page, Bethan; University of Oxford, Experimental Psychology<br>Butler, Siobhan; Helen and Douglas House<br>Smith, Colette; Oxford Health NHS Foundation Trust<br>Lee, Alex CH; Oxford University Hospitals NHS Foundation Trust<br>Vincent, Charles A; University of Oxford, Experimental Psychology
Keywords:	Gastroenterology, Health services research, Nursing

SCHOLARONE™
Manuscripts

I, the Submitting Author has the right to grant and does grant on behalf of all authors of the Work (as defined in the below author licence), an exclusive licence and/or a non-exclusive licence for contributions from authors who are: i) UK Crown employees; ii) where BMJ has agreed a CC-BY licence shall apply, and/or iii) in accordance with the terms applicable for US Federal Government officers or employees acting as part of their official duties; on a worldwide, perpetual, irrevocable, royalty-free basis to BMJ Publishing Group Ltd ("BMJ") its licensees and where the relevant Journal is co-owned by BMJ to the co-owners of the Journal, to publish the Work in this journal and any other BMJ products and to exploit all rights, as set out in our licence.

The Submitting Author accepts and understands that any supply made under these terms is made by BMJ to the Submitting Author unless you are acting as an employee on behalf of your employer or a postgraduate student of an affiliated institution which is paying any applicable article publishing charge ("APC") for Open Access articles. Where the Submitting Author wishes to make the Work available on an Open Access basis (and intends to pay the relevant APC), the terms of reuse of such Open Access shall be governed by a Creative Commons licence – details of these licences and which Creative Commons licence will apply to this Work are set out in our licence referred to above.

Other than as permitted in any relevant BMJ Author's Self Archiving Policies, I confirm this Work has not been accepted for publication elsewhere, is not being considered for publication elsewhere and does not duplicate material already published. I confirm all authors consent to publication of this Work and authorise the granting of this licence.

Confidential: For Review Only

Training and support for caring for a child's gastrostomy:

A survey with family carers

Confidential: For Review Only

Bethan Page[1], Siobhan Butler[2], Colette Smith[3] Alex CH Lee[4], Charles A Vincent[1]

1 Department of Experimental Psychology, New Radcliffe House, Radcliffe Observatory Quarter, Woodstock Road, Oxford, OX2 6AE, UK

2 Helen & Douglas House, Oxford, UK

3 Oxford Health NHS Foundation Trust, UK

4 Oxford University Hospitals NHS Foundation Trust, UK

Correspondence to

Bethan Page, Department of Experimental Psychology, University of Oxford, New Radcliffe House, Radcliffe Observatory Quarter, Oxford, UK, OX2 6GG, Bethan.page@psy.ox.ac.uk

WORD COUNT: 3296

ABSTRACT

Objective

The aim of this study was to explore family carers' experiences of training and ongoing support for caring for their child's gastrostomy, and to get their views on how this could be improved.

Methods

A mixed-methods online survey with 146 family carers (e.g. parents, grandparents) who care for a child with a gastrostomy. Family carers rated their own experience of training and support and made recommendations for how training and support could be improved for future families.

Results

The nature and extent of the training family carers reported receiving varied considerably. Many felt that the demonstrations they received in hospital were too brief. Two in five family carers rated their confidence caring for their child's gastrostomy as very low in the first few weeks after surgery. Parents valued ongoing learning and support from other parents and support from community nurses. Videos and simulation practice were rated as useful formats of training, in addition to face-to-face supervised practice with a clinician. Parents liked how real life the example video shown was, and rated nearly all suggested video topics as "very helpful", especially troubleshooting topics.

Conclusions

Our study found substantial variability in family carers' descriptions of the training and support they received to care for their child's gastrostomy. Training often did not meet family carers' needs. We need to invest in better training and support for families and learn from their recommendations. Improvements to training and support for families (e.g. through instructional videos) have the potential to improve family carers' confidence and competence, and reduce the risk of problems and complications which cause harm to children and increase demand on NHS resources.

Confidential: For Review Only

What is already known on this topic

- It is now commonplace for families to carry out complex medical procedures for children at home.
- Some studies have reported safety concerns around the practices of parents caring for children with gastrostomies.

What this study adds

- The training family carers report receiving to care for their child's gastrostomy varies considerably and often does not fully meet their needs.
- Many family carers report feeling anxious and under-confident in the first few weeks at home after their child's surgery.
- Family carers value supervised practice with healthcare professionals, videos featuring parents and clinicians and more hands on practice, including using models/dolls.
- Training should begin prior to the hospital admission for surgery and be regarded as an essential component of the care package.

Confidential: For Review Only

INTRODUCTION

Many medical procedures, which were once carried out only by healthcare professionals on hospital wards, are now performed by families at home.[1-3] There are increasing numbers of children with complex medical needs.[4,5] Their parents perform a range of medical procedures for them at home, including feeding tube care, home oxygen, tracheostomy care and bowel washouts.[1,4,6] There are numerous benefits for families and the healthcare system when children can be cared for at home, rather than in healthcare settings,[7,8] but there are also substantial risks.[2] An analysis of incident reports on enteral tube feeding in the United Kingdom (UK) identified various safety concerns relating to training and support for parents.[9] Without consistently high-quality training and support for families, children are at risk of harm, and families feel under-confident, anxious and alone.[6,10]

Caring for a child with a gastrostomy

A gastrostomy is a surgically placed device where feeds are delivered directly into the stomach. Gastrostomies are common in children with severe chronic illnesses and neurodisability who have difficulty swallowing or cannot get adequate nutrition through eating and drinking.[11,12] Family carers (e.g. parents) learn to administer feeds, water and medications via the tube, to clean and care for the stoma site, and to manage complications such as leakage and blocked tubes. Family carers may also learn to change a gastrostomy button. Family carers normally receive some bedside teaching in hospital (during a hospital stay of around two days), with ongoing support from community nurses, but this may not be sufficient to ensure safe care at home. There are some safety concerns reported in the literature around the practices of parents caring for children with gastrostomies.[9,13,14] In a recent study on same-day discharge for gastrostomy surgery, the most common reason for a delayed discharge was parents not feeling sufficiently confident caring for the gastrostomy.[15] Improving training and support for families could reduce callouts to over-stretched community teams, as well as preventing avoidable Emergency Department visits and admissions, e.g. through appropriate timely management of a dislodged button.

Many parents caring for children with complex medical needs develop substantial expertise over time.[6,9,10] However training for families can be informal and highly variable. [6,10,11] This contrasts with training for healthcare professionals who receive years of university training, practice-based training and ongoing continuing professional development (CPD). Healthcare professionals typically also have on-site backup and support, whereas families are often home alone whilst carrying out medical tasks.[16] Good quality training for family carers is key for optimising outcomes for children.[17] The aim of this study is to explore family carers' experiences of training and ongoing

Confidential: For Review Only

support for caring for their child's gastrostomy, and to find out their views on how training and support could be improved. This paper is a part of a longer-term project to develop a package of training and support for families.[18]

METHODS

Survey aims

The first aim was to understand family carers' experiences of training and support for caring for their child's gastrostomy. The second aim was to understand family carers' recommendations for improving training and support.

Survey development

The survey was developed in stages. In the initial exploratory stage, we reviewed the literature on gastrostomy training and consulted parent representatives, nurses and paediatricians from the community and hospital. The content of the survey was informed by findings from a preliminary qualitative survey with 50 families who performed a range of medical procedures, which highlighted family carers' feelings of being scared and unprepared, the variability in experiences of training and the emotional demands on families.[19] The survey instrument was drafted, piloted and revised in consultation with parent representatives, children's nurses and paediatricians from the community, a paediatric gastrointestinal surgeon and a specialist surgical nurse. The team were asked to comment on the suitability of the questions, readability and length. Recruitment strategies were developed based on advice from our parent representatives, clinicians and charities.

Survey design and content

The survey was a mixed-methods survey with the qualitative data intended to complement, illustrate and expand the quantitative data.[20] The survey tool used was Qualtrics. The survey is available in Supplementary File 1.

Sampling and Recruitment

Participants were recruited through UK charities and local charities and through our parent representatives who posted on closed Facebook groups which serve as support groups for families ('Tube Feeding your child in the UK' which has 4105 members, and the 'Blended Diet UK' group which has 4200 members). The sample is best described as a convenience sample, however we purposely advertised through charities that support children with a range of different diagnoses and levels of complexity (e.g. Well Child, TOFS, Together for Short Lives) and sought to recruit family carers with different levels of experience (assessed as number of years since the child's gastrostomy surgery). It

Confidential: For Review Only

was clear from the survey question ‘where did you hear about the survey’ that some people also chose to share the survey with friends/family. The advertisement information informed participants that we were looking for family carers (e.g. parents) who cared for children with gastrostomies to complete a survey on their training and support needs. The exact wording varied slightly between the different charities and posts on Facebook groups. The first page of the survey gave some brief information about the survey (see Supplementary File 1).

The inclusion criteria were any parent or family carer who provides gastrostomy care at home to a child or young person aged under 25 years. By family carer, we included any unpaid carer (parent, relative, friend) who actively participates in caring for a child with a gastrostomy. To take part family carers needed to be at least 18 years old. Participants received a £10 voucher for taking part. All participants gave informed consent before taking part and consented for their data to be used in publications (The Medical Sciences Inter-Divisional Research Ethics Committee, Oxford University, R56623/RE004). The data were collected between July and September 2020. We aimed to recruit at least 100 participants to capture a broad range of experiences, including family carers new to gastrostomy care and some more experienced.

Analysis

Descriptive statistics were computed for all close-ended questions, using SPSS Statistics 25. Participants who did not fully complete the survey (defined as viewing all pages of the survey and completing all the quantitative questions at a minimum) were excluded. The open-ended questions were coded in NVivo 12 using inductive content analysis, to group responses based on surface level of meaning.[21] Answers were coded line by line, and grouped into categories emerging from the data. These were summarised and illustrated with quotes from participants.

Patient and Public Involvement

Two parent representatives were involved in the design, conduct and dissemination of the research. The two parents attended the research meetings from conception of the project, alongside a team of multidisciplinary healthcare professionals. The recruitment strategy was devised through consultation with our parent representatives. The parents completed the draft survey, which was then revised based on their feedback and feedback from clinicians. The recommendations from the survey were developed through meetings with parents and the healthcare professionals supporting the research.

https://mc.manuscriptcentral.com/bmjpo

Confidential: For Review Only

RESULTS

Participants

One-hundred-and-forty-six participants fully completed the survey. 195 participants consented to take part and 250 responders viewed the first page. The majority of the 146 participants were mothers (91%). There was a range of ages of the children and time since initial gastrostomy surgery. The most common types of devices were gastrostomy buttons and percutaneous endoscopic gastrostomy (PEG) tubes. Table 1 gives more detail about the participants.

Table 1 About the participants who completed the full survey

	N (%)
**Relation to child**	
Mother	133 (91%)
Father	8 (6%)
Other family member	5 (3%)
**Age of participants' children (years)**	
0-4	50 (34%)
5-9	38 (26%)
10-14	39 (27%)
15-19	18 (12%)
20-25	1 (1%)
**Time since initial gastrostomy surgery (years)**	
<1 year	15 (10%)
1-2	41 (28%)
3-4	27 (18%)
5+ years	63 (43%)
**Type of gastrostomy device child has or previously had** *	
PEG tube	73 (50%)
Gastrostomy button (MINI or Mic-Key)	115 (79%)
Another device (e.g. gastro-jejunostomy (GJ) tube)	23 (16%)

* Some children had more than one gastrostomy device, e.g. some children had a PEG tube initially which was later changed to a gastrostomy button.

Participants' descriptions of their training

Participants' experiences of training were variable (see Box 1). Most described receiving some training in hospital but the nature and extent of this training varied considerably. Descriptions of

Confidential: For Review Only

training as "*brief*" or "*basic*" were common. Some participants felt unprepared and anxious: *"It was scary because we were worried about it getting caught/pulled and hurting or causing damage to our little boy. We didn't get much practice before being left to do it on our own so you are triple checking yourself and worrying did I do it right."* Some participants described receiving further training at home by community nurses or representatives from a feeding company, as well as learning through other parents, often through Facebook groups: *"Also had amazing advice from other mums whose children have tubes who I found via Facebook groups - game changing stuff."*

Box 1 Example quotes illustrating the variability in experiences of training

Training sometimes brief & basic:

- *"A brief 5 mins of basics, a leaflet and home. A few weeks later a Nutricia nurse came to the house to show us the pump."*
- *"We were admitted for the surgery and spent two further days in the ward after, received very basic training on the use of the peg and left feeling absolutely terrified about using it!"*
- *"Need much more training than 10 minutes in consultant's office!"*

Primarily self-taught with support from other parents:

- *"The official training - nothing good. Absolutely disastrous, vague, unsupportive. The unofficial training and the info I sought out for myself - clear, helpful videos from other parents, useful approach of gastrostomy nurse. Properly child-centric, helpful stuff."*

More community support needed:

- *"The community nursing team rely too much on the surgical nurse to do the training and then they just catch up with a chat following any training given at the bedside. This training is not detailed/long enough for those dealing with such a complex medical needs child."*

Thorough training and support received:

- *"Our daughter was in hospital long terms due to a range of factors. We were able to observe nurses undertaking feeds, using pump, giving medication. We were also given an information pack and work book to go through, and we were observed by nurses until confident and competent to do ourselves. We were given support in community… with regards to changing button, taking care of button, and annual refresher also. Received updates with regards to how much water to inflate balloon with, frequency of Ph testing etc."*

The majority of participants reported receiving some training from their hospital team (n=115, 79%). A slightly smaller proportion received training from a Community Children's Nurse (CCN) (n=105, 72%). 35 participants (24%) mentioned training from another provider, most commonly a feeding company (e.g. Nutricia). 82 (56%) participants reported receiving training from both the hospital and CCN team, and 8 (5%) reported not receiving training from either the hospital team or a CCN team.

Table 2 shows the types of training participants received. The most common types of training were verbal information, demonstrations and supervised practice from a healthcare professional. Few participants received any simulation practice (hands on practice with a doll/equipment), or watched instructional videos. A few of the participants that did report receiving simulation practice commented on the usefulness of this: *"Really useful being able to have a little play with a tube and practice using the clip etc. before having to do it for real."*

Table 2 Types of teaching/training received by parents

Types of teaching/training received	N (%)
Given information verbally by a healthcare professional	129 (88%)
Demonstrations by a healthcare professional	125 (86%)
Practised on my child supervised by a healthcare professional	113 (77%)
Given a written booklet	85 (58%)
Simulation practice (practiced with a doll or some equipment)	19 (13%)
Directed to a website for information	13 (9%)
Demonstrations by another family member	7 (5%)
Other	5 (3%)
Videos	3 (2%)

Confidence over time

Participants were asked how confident they felt caring for their child's gastrostomy in the first few weeks after surgery: 24 (16%) were not at all confident, 32 (22%) were slightly confident, 30 (21%) were moderately confident, 42 (29%) were mostly confident and 18 (12%) of participants said they were fully confident. At the time of the survey a majority (n=117, 80%) said they felt fully confident caring for their child's gastrostomy. The most common concern participants reported were around hurting their child, caring for site after surgery, knowing "*what was normal*" in relation to the stoma site healing, worries about the tube coming out and problems such as blocked tubes or granulation:

https://mc.manuscriptcentral.com/bmjpo

Confidential: For Review Only

"The first time I experienced this [granulomas] I thought my sons intestines were coming out! Nobody had ever mentioned it to me nor had I ever seen anything like it!"

It is not possible to say from the data whether the training participants received had improved over time. However, we did not find any evidence suggesting that participants' retrospective confidence ratings from the first week at home had changed over time, which may suggest that training has not changed or improved over time: 40% of participants with less than a year's experience rated themselves as 'not at all confident' or 'slightly confident' in the first week at home, compared to 38% of participants with more than 5 years' experience. There was no statistically significant association between time since gastrostomy surgery and participants' ratings of confidence in the first week at home: $\chi^2(12, N=147) = 12.06$, $p = .44$. Again, this may suggest that training has not changed or improved over time.

However, there was evidence that participants' ratings of their current confidence (at the time of the survey) did improve with more years' experience: 46% of participants with less than a years' experience rated themselves as fully confident caring for their child's gastrostomy compared to 89% of participants with more than 5 years' experience. A chi-squared test revealed a significant association between current confidence ratings and number of years since gastrostomy surgery: $\chi^2(9, N=147) = 17.54$, $p = .04$.

Ongoing support and training

Participants were asked which sources were most helpful for ongoing support (see Table 3). Contacting your community nurse was rated as "very helpful" by a majority, with less than a quarter rating hospital teams as a very helpful source of support. Notably conversations with other parents was rated as "very helpful" by 56%, with a further 32% rating support from parents as "quite helpful". Participants in the survey offered advice for other parents, including tips for managing their child's distress when changing a gastrostomy button.

Table 3 Sources of ongoing support: how helpful are they?

	Very helpful	Quite helpful	Not very helpful	Not applicable
Contacting your community nurse	90 (62%)	28 (19%)	13 (9%)	15 (10%)
Contacting your hospital team	34 (23%)	37 (25%)	38 (26%)	37 (25%)
Facebook groups	54 (37%)	67 (46%)	11 (8%)	14 (10%)
Conversations with other parents of children with similar needs	82 (56%)	47 (32%)	3 (2%)	14 (10%)

https://mc.manuscriptcentral.com/bmjpo

Written information booklets provided by a healthcare professional	28 (19%)	68 (47%)	32 (22%)	18 (12%)
NHS websites	15 (10%)	55 (38%)	52 (36%)	24 (16%)
Charities	25 (17%)	53 (36%)	19 (13%)	49 (34%)
Videos	29 (20%)	50 (34%)	17 (12%)	50 (34%)

Participants were asked, at the time of completing the survey, whether further training might be helpful to them: 14 (10%) said definitely yes to further training or support, 29 (20%) probably yes, 81 (55%) probably not and 21 (14%) definitely not. The most common request was help with managing problems. A number of participants wanted refresher training, or updates on the latest guidance: *"making sure bad habits have not crept in and that we are up-to-date with any changes in how things are done."*

Participants' recommendations for improving training

Participants were asked which formats of training might be useful to other parents facing the challenge of caring for a child with a gastrostomy (see Table 4). Around three-quarters rated demonstrations and practice on your child supervised by a healthcare professional as extremely useful. Around two-thirds of participants felt that videos and simulation practice would be extremely or very useful. Participants felt that these additional forms of training would be a useful addition to face-to-face training with a professional, but not a substitute: *"I think that the videos are good resources that parents can go back to however I think that face to face training is really important initially."*

Table 4 Which types of training might be helpful to other parents

	Extremely useful	Very useful	Moderately useful	Slightly useful	Not at all useful
Demonstrations by a healthcare professional	105 (72%)	33 (23%)	6 (4%)	2 (1%)	0 (0%)
Practised on my child supervised by a healthcare professional	110 (75%)	29 (20%)	5 (3%)	1 (1%)	1 (1%)
Written booklets	32 (22%)	39 (27%)	51 (35%)	19 (13%)	5 (3%)
Videos	40 (27%)	58 (40%)	29 (20%)	14 (10%)	5 (3%)
Simulation training (practicing with a doll or some equipment)	47 (32%)	45 (31%)	36 (25%)	10 (7%)	7 (5%)

Confidential: For Review Only

Online training	18 (12%)	33 (23%)	47 (32%)	30 (21%)	17 (12%)
Group training with other parents	20 (14%)	34 (23%)	39 (27%)	31 (21%)	21 (14%)
Training by experienced parents	15 (10%)	16 (11%)	32 (22%)	51 (35%)	32 (22%)

Confidential: For Review Only

Participants had many suggestions for improving training. A common suggestion was more training and information about common problems including what to do if the button comes out, and requests for specific types of training, such as more hands on training. Some participants commented on the timing of training as they struggled to take information on board while in hospital: *"I think we should have had more [training] before his surgery and not while he was in theatre as we were so anxious I'm not sure how much we took in or how valuable doing it then was."* A few participants said they would like to have been put in touch with other families: *"Opportunity to do group talks or training would be good. Nice way to meet other families and build a support network."*

Recommendations for developing training videos

Participants watched a sample video showing a parent administering a bolus of water to their child. Participants liked how "real life" the video was: the child was wriggling during the procedure, it was done at home, it was relaxed and the mother was talking to the child throughout. One participant commented that: "*I've hated the teaching videos previously as they seem rather clinical but this was fantastic*." When asked where training videos should be filmed, 54 (37%) said at home, 4 (3%) said in hospital, and 88 (60%) said a mixture of both locations. 116 (80%) of participants wanted both healthcare professionals and parents to feature, 12 (18%) wanted just healthcare professionals and 8 (12%) wanted just parents. Participants rated different topics for videos: the vast majority were rated as 'very helpful' (see Supplementary File 2). More specific recommendations from participants for developing training videos are available in Supplementary File 2.

DISCUSSION

Family carers' descriptions of the training they received to care for their child's gastrostomy varied considerably. Demonstrations in hospital were frequently described as too brief and insufficient to prepare families. Many family carers reported feeling anxious in the first few weeks of caring for their child at home and concerns about doing something wrong or hurting their child. Most family carers however reported feeling confident to care for their child's gastrostomy at the time of the survey. Family carers particularly valued ongoing support from other parents and from community

nurses. Videos and simulation practice were rated as useful preparation, in addition to face-to-face supervised practice with a healthcare professional. Participants wanted videos to feature parents and healthcare professionals and for at least some of the videos to be filmed at home.

Strengths and limitations

This study has a number of strengths. Parents with different levels of experience responded to the survey, from families who were very new to gastrostomy care to families with more than 5 years' experience. Families were recruited through charities and Facebook support groups whose members come from across the UK: this suggests that the issues described by families in the survey are not unique to one region or service. Families were very engaged and many offered to help support the development of training videos and other resources.

One key weakness of the study is that we did not collect demographic data on the families so cannot tell the socio-economic, health literacy or ethnicity of families, or information about the children's diagnoses or their level of medical complexity. It is therefore impossible to know how selection bias played out in our study. We may have recruited families who are more engaged in their child's care or families who felt unprepared and sought help through Facebook groups and charities. It is possible there are some issues of recall in family carers who received their training a long time ago (43% of parents had more than 5 years' experience of caring for their child's gastrostomy).

Implications for the design of services

Good quality training for family carers is recognised in the literature as key for optimising outcomes for children and preventing harm.[14,17] Shorter hospital stays are increasingly advocated for gastrostomy surgery, including same day discharges,[15] meaning that there is little time to train families during admission. Many family carers in our survey reported not feeling confident caring for their child in the first few weeks at home after surgery. Other studies have similarly reported concerns that some families do not feel confident to care for their child's gastrostomy upon discharge from hospital,[15] as well as gaps in knowledge and inadequate skills in some family carers.[13,14] Our study documents some of family carers' concerns and also importantly their recommendations for improving training and support. Family carers may benefit from more preparation for caring for their child's gastrostomy (from either hospital or community nurses) before surgery takes place: this has the potential to improve confidence ratings in the first few weeks at home after surgery.

Box 2 gives an overview of practical suggestions for improving training based on discussions of the survey data with our multidisciplinary group of healthcare professionals and parents. Family carers

https://mc.manuscriptcentral.com/bmjpo

may benefit from viewing instructional videos and written materials before the surgery with the resources available to revisit as needed. To enable more opportunity for hands-on practice, family carers could practice with dolls and equipment: there is substantial evidence on the benefits of repeated hand-on-practice in the medical education literature.[22,23] There is potential to make more use of online training and support, including group video-calls for families. Training resources should be co-designed with families and address their emotional needs (such as recognising parental anxiety and fears and discussing the potential impact of a gastrostomy on daily life) as well as the technical aspects of care.

Box 2: Practical suggestions for improving training based on discussions on the survey findings with a multidisciplinary group of healthcare professionals and parents.

Before surgery

- Family carers may benefit from videos teaching them about routine care of a gastrostomy, how to manage common problems, and advice and tips from more experienced families. Families recommend videos should feature both parents and healthcare professionals and some should be filmed at home. It is also important for the videos to feature families from different cultural backgrounds and to be accessible to families who do not speak fluent English.
- Family carers may benefit from a home visit (e.g. from a community nurse) for face-to-face teaching and the opportunity to ask questions.
- Family carers may value repeatedly practicing with dolls/equipment to become familiar with the basics before their child's surgery, e.g. connecting the extension tube.
- Family carers could be invited to a group call with other families awaiting surgery to ask questions to the surgical team and meet other parents.
- Family carers may benefit from recommendations for Facebook groups to join and other peer support options.

During hospital admission

- Family carers highly value supervised practice doing procedures on their child after surgery with the support of a designated nurse. It is important that adequate time is given to this important part of training.

After hospital admission

- Family carers will likely benefit from further support from community nurses who can provide further teaching and support through home visits and video-calls.
- Family carers will likely benefit from revisiting videos and written materials as needed, including videos on managing common problems such as overgranulation or blocked tubes.
- Family carers need to be supported by community nurses to learn to change a button, and may benefit from repeated practice with models in addition to supervised changes on their child.

Future research and development

https://mc.manuscriptcentral.com/bmjpo

Our multidisciplinary group is currently creating a library of videos co-produced with families, paediatricians and nurses from the hospital and community and piloting hands-on practice with equipment and 3D-printed models. The videos and further information about the programme are available online.[18] Evaluation of the videos is ongoing. Future research is needed to better understand healthcare professionals' views on training families and ideas for improvement, to compare against the findings from the families in this survey. It is also important to explore the experiences of training in family carers with low health literacy or with limited English language.

CONCLUSIONS

Our study found substantial variability in family carers' descriptions of the training and support they received to care for their child's gastrostomy. Many felt that the training they received did not prepare them sufficiently. Family carers valued face-to-face training with a healthcare professional, videos which show "real-life" featuring families and clinicians and hands-on practice including with dolls/equipment. We need to invest in better training and support for families and learn from their recommendations. Improvements to training and support for families have the potential to improve family carers' confidence and competence, and reduce the risk of problems and complications which cause harm to children and increase demand on NHS resources.

Confidential: For Review Only

REFERENCES

1. Ten Haken I, Ben Allouch S, Van Harten WH. The use of advanced medical technologies at home: A systematic review of the literature. *BMC Public Health* 2018;**18**(1):284.

2. Institute for Healthcare Improvement. No Place Like Home: Advancing the Safety of Care in the Home 2018. Available from: http://www.ihi.org/resources/Pages/Publications/No-Place-Like-Home-Advancing-Safety-of-Care-in-the-Home.aspx

3. Reinhard S, Levine C, Samis S. Home alone: family caregivers providing complex care. *Gerontologist* 2017;**56**(Suppl_3):636–636.

4. Cohen E, Berry JG, Sanders L, Schor EL, Wise PH. Status Complexicus? The Emergence of Pediatric Complex Care. *Pediatrics* 2018;**141**(Suppl 3):S202–S211.

5. Fraser L, Gibson-Smith D, Jarvis S, Norman P, Parslow R. "Make Every Child Count" Estimating current and future prevalence of children and young people with life-limiting conditions in the United Kingdom. 2020. Available from: https://www.togetherforshortlives.org.uk/resource/make-every-child-count/

6. Page BF, Hinton L, Harrop E, Vincent C. The challenges of caring for children who require complex medical care at home: 'The go between for everyone is the parent and as the parent that's an awful lot of responsibility.' *Heal Expect* 2020;**23**(5):1144-1154.

7. Glasson EJ, Forbes D, Ravikumara M, Nagarajan L, Wilson A, Jacoby P, et al. Gastrostomy and quality of life in children with intellectual disability: A qualitative study. *Arch Dis Child* 2020, **105**(10):969-974.

8. Mitchell TK. The impact of medical technology upon the home and feelings of at-homeness for children and young people with complex health care needs and their family members. PhD Thesis Tracy Karen Mitchell, Edge Hill University. 2020. Available at: https://research.edgehill.ac.uk/en/studentTheses/the-impact-of-medical-technology-upon-the-home-and-feelings-of-at

9. Page B, Nawaz R, Haden S, Vincent C, Lee ACH. Paediatric enteral feeding at home: An analysis of patient safety incidents. *Arch Dis Child* 2019;**104**:1174-1180.

10. Spiers G, Beresford B. "It goes against the grain": A qualitative study of the experiences of parents' administering distressing health-care procedures for their child at home. *Heal Expect* 2017; **20**(5):920-928.

https://mc.manuscriptcentral.com/bmjpo

Confidential: For Review Only

11. Burdall OC, Howarth LJ, Sharrard A, Lee ACH. Paediatric enteral tube feeding. *Paediatrics and Child Health* 2017; **27**(8):P371-377,2017.

12. Puntis JWL. Benefits and management of gastrostomy. *Paediatrics and Child Health* 2009;**19**(9):415–24.

13. Evans S, Shelton F, Holden C, Daly A, Hopkins V, MacDonald A. Monitoring of home safety issues in children on enteral feeds with inherited metabolic disorders. *Arch Dis Child* 2010; **95**(9): 668–672.

14. Evans S, Preston F, Daly A, Ashmore C, MacDonald A, Holden C. Home enteral tube feeding in children with inherited metabolic disorders: a review of long-term carer knowledge and technique. *J Hum Nutr Diet* 2012;**25**(6):520-525.

15. Dekonenko C, Svetanoff WJ, Osuchukwu OO, Pierce AL, Orrick BA, Sayers KL, et al. Same-day discharge for pediatric laparoscopic gastrostomy. *J Pediatr Surg* 2020; **56**(1):26-29.

16. Reinhard, S., Levine, C., & Samis, S. (2012). HOME ALONE: Family Caregivers Providing Complex Chronic Care. In *AARP Public Policy Institute*.

17. Berman L, Hronek C, Raval M V, Browne ML, Snyder CL, Heiss KF, et al. Pediatric gastrostomy tube placement: Lessons learned from high-performing institutions through structured Interviews. *Pediatr Qual Saf* 2017;**2**(2):e016.

18. Oxford Simulation Teaching and Research Centre. Supporting Parents. 2021. https://www.oxstar.ox.ac.uk/more/supporting-parents (accessed 6th April 2021)

19. Page B. Development of training and support for parents who perform specialist medical care at home. DPhil Thesis. Bethan Francesca Page, University of Oxford. 2021 [Unpublished].

20. Terry G, Braun V. Short but Often Sweet. In: Collecting Qualitative Data. Cambridge University Press; 2017.

21. Elo S, Kyngäs H. The qualitative content analysis process. *J Adv Nurs* 2008;**62**(1):107–15.

22. Motola I, Devine LA, Chung HS, Sullivan JE, Issenberg SB. Simulation in healthcare education: A best evidence practical guide. AMEE Guide No. 82. *Med Teach* 2013;**35**(10):142–59.

23. McGaghie WC. Does Simulation-based Medical Education with Deliberate Practice Yield Better Results than Traditional Clinical Education? A Meta-Analytic Comparative Review of the Evidence. *Acad Med* 2012;**86**(6):706–11.

Confidential: For Review Only

Acknowledgements

We would like to thank all the parents who completed the survey. We are grateful for the expertise of our multidisciplinary stakeholder group, which includes parents, paediatricians, children's nurses and specialist surgical nurses. We thank the team for their ongoing work developing training for parents. This work was supported a grant from Health Foundation's Q Exchange Programme. BP is supported by the Economic and Social Research Council [grant number ES/J500112/1]. CV is supported by the Health Foundation.

Contributorship statement

BP and CV conceived and designed the study. The survey was designed and revised by BP with support from SB, CS, AL & CV. BP analysed the data. BP drafted the manuscript with support from SB, CS and CV. All authors provided critical feedback on the manuscript and analysis. All authors approved the final manuscript.

Competing interests

None declared.

Data sharing statement

Data may be obtained upon reasonably request by contacting the corresponding author. The survey tool used is available in the supplementary information.

Confidential: For Review Only

Supplementary File 1: Survey Tool

Overview of survey

Gastrostomy care: an online survey for parents on training and support needs

- This survey is for family carers (e.g. mums, dads, grandparents) who care for a child or young person who has a gastrostomy (e.g. PEG or button).
- This survey is part of a project to develop resources to better prepare and support families who are new to gastrostomy care. The project is a collaboration between researchers, parents and healthcare professionals.
- The findings will be shared widely with parents, charities, healthcare providers and researchers.

What do I have to do to take part?

- This study is an online survey and will take 15-20 minutes to complete.
- You will be asked about your experiences of learning to care for your child's gastrostomy, your experiences of ongoing support and what videos and resources might be helpful.
- To take part, the child you care for needs to be under 18 years and you need to be at least 18 years old.
- Please read the participant information sheet on the next page if you are interested in taking part.

Thank you for your time.

Page Break

Confidential: For Review Only

Participant information

We [the University of Oxford in collaboration with clinicians and parent representatives] are investigating the experiences and training needs of family carers in the UK who provide specialist healthcare to children at home, in order to develop resources for training and supporting other families. We are currently creating videos and resources for parents new to gastrostomy care and are seeking your feedback on how we should do this.

We appreciate your interest in participating in this online survey. You have been invited to participate as you are a parent or a family carer who provides gastrostomy care to a child or young person under 18 at home. By family carer, we include any unpaid carer (parent, relative, friend, foster carer) who actively participates in caring for a child or young person with a gastrostomy. Tasks you might do include giving feeds, medications, caring for the stoma site and/or changing a gastrostomy button. To take part you, the family carer, need to be at least 18 years old.

Please read through this information before agreeing to participate by ticking the 'yes' box below.

You will be asked questions on:

i) you and your child (e.g. child's age, your relationship to the child)

ii) your experiences of learning and being trained to care for your child's gastrostomy

iii) your experiences of ongoing support with caring for your child's gastrostomy

iv) what training and support would help other parents

v) what videos would be helpful

The survey should take 15-20 minutes to complete. No background knowledge is required.

Confidential: For Review Only

How will your data be used?

The survey will be used to inform the development of videos and other resources to support and prepare families new to gastrostomy care. Some of the videos will also be helpful to more experienced parents and for training staff.

The study results will be written up in a DPhil (PhD) thesis and potentially also in an academic publication. Key findings will be shared widely with participants, family carers, charities, health care professionals and researchers.

Your answers will be completely anonymous, and we will use all reasonable endeavours to keep them confidential. You will be given the option at the end of the survey to provide us with your email address if you would like to receive a summary of the research findings or if you would be interested in helping us rate and evaluate the videos we are creating. Email addresses and telephone numbers will be stored on a password protected file on a secure university server. This is part of a longer term project to develop new methods for training and supporting family carers.

Please note that your participation is voluntary. You may withdraw at any point during the survey for any reason, before submitting your answers, by closing the browser. If you need to leave the survey before you have time to fully complete it, you can return to finish your partially completed survey using the same device (tablet, computer, mobile) within one week of starting it. After one week, your data will be saved and you will be unable to continue the survey.

Your data will be stored in a password-protected file on secure university servers and may be used in academic publications. Your IP address will not be stored. All questions are optional.

Who will have access to your data?

The University of Oxford is the data controller for the purposes of the Data Protection Act 1998. Your information may be shared with members of the research team. This questionnaire is for a DPhil [PhD] project. The principal researcher is Bethan Page, who is attached to the Department of Experimental Psychology at the University of Oxford. This project is being completed under the supervision of Professor Charles Vincent, Professor Nick Yeung and Dr Emily Harrop (clinical lead for the project).

Confidential: For Review Only

This project has been reviewed by, and received ethics clearance through, the University of Oxford Central University Research Ethics Committee [reference number R56623/RE001].

Participant benefits and risks

If you complete the full survey, we will email you a £10 Marks and Spencer (M&S) voucher as a thank you for taking part. You will be asked to leave your email address at the end of the survey so we can send you the voucher. Your email address will be deleted from our records after we have sent you the voucher (unless you have consented to be contacted in the future with a summary of the findings, or to help with future studies).

There will be no other direct benefits to you in taking part in this study. However, you may find it useful to share your experiences and views on improving training and support. Your involvement will improve our understanding of current training provision and support needs of family carers. Key findings will be used to develop resources to better support and prepare family carers, and will be shared widely with families, healthcare providers, charities and researchers.

There are no direct risks involved in taking part in this study. However it is possible that you may find some of the topics distressing or difficult. You don't have to answer all the questions and you can exit the survey at any point or withhold any information that you do not wish to share. A list of website and charities offering further support will be provided at the end of the survey.

What if there is a problem?

If you have a concern about any aspect of this project, please contact the researcher Bethan Page (email: bethan.page@psy.ox.ac.uk) or their supervisor Professor Charles Vincent (charles.vincent@psy.ox.ac.uk) who will do their best to answer your query. If your problem is a clinical query, we will contact the clinical lead for the project, Dr Emily Harrop (Consultant in Paediatric Care) for guidance.

The researcher should acknowledge your concern within 10 working days and give you an indication of how they intend to deal with it. If you remain unhappy or wish to make a formal

Confidential: For Review Only

complaint, please contact the relevant Chair of the Research Ethics Committee at the University of Oxford:

Chair, Medical Sciences Inter-Divisional Research Ethics Committee;

Email: ethics@medsci.ox.ac.uk;

Address: Research Services, University of Oxford, Wellington Square, Oxford OX1 2JD

The Chair will seek to resolve the matter in a reasonably expeditious manner.

If you have any questions about the survey please email bethan.page@psy.ox.ac.uk.

Please note that you may only participate in this survey if you are 18 years of age or over.

- ○ I am 18 years or over

If you have read the information above and agree to participate with the understanding that the data (including any personal data) you submit will be processed accordingly, please check the relevant box below to get started.

- ○ Yes I agree to take part

Confidential: For Review Only

About you and your child

Section 1 of 5:
In this first section we would like to ask some questions about you and the child or young person you provide care for.

Q1.1 What is your relationship to the child that you care for?

- ◯ Mother (1)
- ◯ Father (2)
- ◯ Other [please specify] (4) ____

Q1.2 How old is your child?

____

Q1.3 How long ago did your child first have gastrostomy surgery?

- ◯ Less than a year (1)
- ◯ 1 - 2 years (2)
- ◯ 3 - 4 years (3)
- ◯ 5 + years (4)

Confidential: For Review Only

Q1.4 Which types of gastrostomy tube has your child had? [select all that apply]

- ☐ Gastrostomy button (e.g. MIC-KEY button, MINI button) (1)
- ☐ Percutaneous Endoscopic Gastrostomy (PEG) (e.g. Freka, Corflo) (2)
- ☐ Other [please specify] (3) ____

Experiences of learning and being trained

Section 2 of 5:
In this section we would like to understand what training and information you received to care for your child's gastrostomy and what you found helpful.

Parents have to learn to give feeds and medications, clean and care for the stoma site and learn what to do if there is a problem, such as if the PEG tube blocks or the gastrostomy button comes out. There are also other tasks you may have learnt to do such as changing a gastrostomy button.

Q2.1 Tell us about what training and information you received when your child first had gastrostomy surgery.

____

____

____

____

____

Q2.2 Tell us about any training or information you have received since the initial surgery.

____

____

https://mc.manuscriptcentral.com/bmjpo

Confidential: For Review Only

____
____
____

Q2.3 Who have you received training or information from? [select all that apply]

- ☐ Hospital team (1)
- ☐ Community Children's Nurse (CCN) (2)
- ☐ Other [please specify] (3) ____

Q2.4 What type of training/teaching have you received? [select all that apply]

- ☐ Given information verbally by a healthcare professional (23)
- ☐ Demonstrations by a healthcare professional (24)
- ☐ Practised on my child supervised by a healthcare professional (32)
- ☐ Demonstrations by another parent or family member (39)
- ☐ Given a written booklet (33)
- ☐ Directed to a website for information (34)
- ☐ Simulation practice (e.g. practised with a doll or some equipment) (35)
- ☐ Videos (38)
- ☐ Other [Please state] (37) ____

Confidential: For Review Only

Q2.5 What was good about the training you received to care for your child's gastrostomy?

____
____
____
____
____

Q2.6 What could have been improved about your training or was there anything missed?

____
____
____
____
____

Q2.8 How confident did you feel to care for your child's gastrostomy **in the first week** at home after their surgery?

- ◯ Not at all confident (1)
- ◯ Slightly confident (2)
- ◯ Moderately confident (3)
- ◯ Mostly confident (4)
- ◯ Fully confident (5)

Confidential: For Review Only

Q2.9 What were you not confident with?

____

____

____

____

____

Experiences of ongoing support

Section 3 of 5:
In this section we would like to understand how confident you feel now and what ongoing support you have found helpful or might want.

Q3.1 How confident do you feel **now** caring for your child's gastrostomy?

- ◯ Not at all confident (1)
- ◯ Slightly confident (2)
- ◯ Moderately confident (3)
- ◯ Mostly confident (4)
- ◯ Fully confident (5)

Confidential: For Review Only

Q3.2 Would it be helpful to receive any further training or support?

- Definitely yes (1)
- Probably yes (2)
- Probably not (3)
- Definitely not (4)

Q3.3 If yes, what would you like further training or support with?

____
____
____
____
____

Confidential: For Review Only

Q3.4 How helpful do you find the following sources for ongoing information and support? (Please select all that apply)

	Very helpful	Quite helpful	Not very helpful	Not applicable
Contacting your community nurse	○	○	○	○
Contacting your hospital team (e.g. specialist nurse)	○	○	○	○
Facebook groups	○	○	○	○
Conversations with other parents of children with similar needs	○	○	○	○
Written information/booklets provided by a healthcare professional	○	○	○	○
NHS websites	○	○	○	○
Charities	○	○	○	○
Videos	○	○	○	○
Other [please state]	○	○	○	○

Confidential: For Review Only

Section 4 of 5: *We are working to develop training and resources for parents new to gastrostomy care.* In this section we would like to get your views on what training and support is most helpful to parents generally.

Q4.1 Which formats for training do you think are most useful?

	Extremely useful	Very useful	Moderately useful	Slightly useful	Not at all useful
Demonstrations by healthcare professionals	○	○	○	○	○
Practice supervised by a healthcare professional	○	○	○	○	○
Written booklets	○	○	○	○	○
Videos	○	○	○	○	○
Simulation training (e.g. hands on practice with a doll or some equipment)	○	○	○	○	○
Online training	○	○	○	○	○
Group training sessions with other parents	○	○	○	○	○
Training by an experienced parent	○	○	○	○	○

Confidential: For Review Only

Q4.2 If you use Facebook groups for ongoing support relating to your child's gastrostomy, are there any you would recommend to other parents? If yes, please name.

Q4.3 We know that some children and parents find some tasks distressing, such as changing a gastrostomy button. Do you have tips to share with parents, or know of any resources that might be helpful to other parents?

Section 5 of 5: *We are developing some training videos for parents who are new to gastrostomy care. Some of the videos may also be helpful to more experienced parents and for training staff. In this final section we would like to get your views on what these videos should look like.*

Q5.1 We have started creating some videos. Here is an example video:

Confidential: For Review Only

Q5.2 What did you like about the video?

Q5.3 What could be improved or was missing in the video?

Confidential: For Review Only

Q5.4 Which of the following videos do you think would be helpful to other parents?

	Really helpful	Quite helpful	Not very helpful	Not applicable to me
Caring for the stoma/gastrostomy site	○	○	○	○
Flushing the tube	○	○	○	○
Giving a feed	○	○	○	○
Giving medications	○	○	○	○
Venting (letting air or gas out)	○	○	○	○
Changing the water in the balloon of the gastrostomy button	○	○	○	○
How to change a gastrostomy button	○	○	○	○
What to do if the tube blocks	○	○	○	○
What to do if the tube comes out	○	○	○	○

Confidential: For Review Only

Q5.5 Some of the videos will cover the technical aspects of care. We also recognise that there are wider topics which parents might find helpful. Which of the suggestions below might be helpful?

	Really helpful	Quite helpful	Not very helpful
Recommendations for Facebook groups to join	○	○	○
Tips for managing your child's distress, e.g. when changing a button	○	○	○
Advice for new parents from more experienced parents	○	○	○

Q5.6 Do you have any other suggestions for topics or things we should consider?

____

____

____

____

____

Q5.7 Where should the videos be filmed?

○ Home

○ Hospital

○ Mixture

Confidential: For Review Only

Q5.8 Who should be delivering the teaching/information in the videos?

- ◯ Parents
- ◯ Healthcare professionals
- ◯ Mixture

Q5.9 Do you have any final comments for us on videos or other resources to support parents?

____

____

____

____

____

Thank you very much for your time today. Your thoughts and advice will be really helpful to us as we develop resources for preparing and supporting families.

Do you have any final comments for us?

____

____

____

____

____

As a thank you for your help we will be sending all participants who complete the full survey a voucher (£10 M&S e-giftcard). Please leave your email address here so we can send it to you (we will aim to email it to you within two weeks).

____

Confidential: For Review Only

Would you like to be emailed a summary of the findings from this project?

- ◯ Yes
- ◯ No

We are currently developing more videos for families new to gastrostomy care. The videos will also be used for training staff. We will need help from parents later in the year to rate/evaluate the videos and resources.

Would you be willing to do this? We will compensate you for your time.

- ◯ Yes, please contact me to help rate/evaluate more videos
- ◯ No, please don't contact me about this.

Below is a link to a list of charities and resources which you may find helpful as a source of information and support. If this survey has raised any questions or concerns about the care of your child, please contact a healthcare professional for advice: Charities and other resources

Confidential: For Review Only

Further details about the survey

For all the closed-ended/quantitative questions we used qualtrics' 'request response' option. This function rem was unanswered questions on the page before they could move on to the next page, but it did not force partici ended qualitative questions did not request a response. We included participants who completed all the quanti
The back button was enabled so participants could go back and check their answers.
We did not do any formal checks for unique site visitors. We identified one participant who had completed the email addresses submitted for the vouchers. The participants' second response was excluded.

Confidential: For Review Only

Supplementary File 2: Family carers' recommendations for topics for videos

Participants were asked to rate various topics for videos on a three point scale with 1 indicating 'not very helpful' and 3 indicating 'very helpful'. The vast majority of topics were rated as 'very helpful', with the lowest mean score being 2.45. Topics related to problem solving were generally rated as slightly more helpful than routine care.

Table 1 Participants' rating of which topics would be most helpful to cover in videos

	M (SD)	Very helpful	Quite helpful	Not very helpful	Not applicable
What to do if the tube comes out	2.90 (0.3)	129 (88%)	13 (9%)	1 (1%)	3 (2%)
What to do if the tube blocks	2.86 (0.4)	126 (86%)	17 (12%)	1 (1%)	2 (1%)
How to change a gastrostomy button	2.84 (0.4)	118 (81%)	18 (12%)	2 (1%)	8 (5%)
Venting (letting air or gas out)	2.82 (0.4)	114 (78%)	23 (16%)	1 (1%)	8 (5%)
Changing the water in the balloon of the gastrostomy button	2.79 (0.4)	110 (75%)	27 (18%)	1 (1%)	8 (5%)
Advice for new parents from more experienced parents	2.76 (0.5)	115 (79%)	27 (18%)	4 (3%)	0 (0%)
Caring for the stoma/gastrostomy site	2.72 (0.5)	106 (73%)	37 (25%)	1 (1%)	2 (1%)
Tips for managing child's distress (e.g. when changing button)	2.63 (0.6)	100 (68%)	37 (25%)	8 (5%)	1 (1%)
Giving a feed	2.63 (0.5)	94 (64%)	48 (33%)	3 (2%)	1 (1%)
Giving medications	2.62 (0.5)	93 (64%)	50 (34%)	3 (2%)	0 (0%)
Recommendations of Facebook groups to join for support	2.49 (0.6)	82 (56%)	52 (36%)	11 (8%)	1 (1%)
Flushing the tube	2.45 (0.6)	76 (52%)	57 (39%)	11 (7.6%)	2 (1%)

Confidential: For Review Only

Family carers' suggestions of other topics to include

Family carers had various suggestions for other topics for videos. Common technical topics included blended diet, tips for specific problems (e.g. replacing connector end of Freka peg) and different types of feeding tubes. They also had suggestions relating to wider support needs, including advice on doing daily activities, ways of explaining feeding tubes to children and siblings and reassuring other families that children can still do normal activities: "*Showing people that they can get out and about with their child still. I know parents who return home to feed their child it would be nice to show how a gtube doesn't stop fun*."

Confidential: For Review Only
